# Renal Cell Carcinoma Metastasizing to Oral Soft Tissues: Systematic Review

**DOI:** 10.1055/s-0044-1782202

**Published:** 2024-05-06

**Authors:** Harnisha Vipulkumar Prajapati, Ruchira Shreevats, Sonia Gupta, Harman Sandhu, Jaskirat Kaur, Jasmine Kaur

**Affiliations:** 1 Bhavya Dental Clinic and Implant Centre, Palanpur, Gujarat, India; 2 Primadent Dental Centre, Bangalore, Karnataka, India; 3 Department of Oral Pathology and Microbiology & Forensic Odontology, Yamuna Institute of Dental Sciences and Research, Yamunanagar, Haryana, India; 4 Building Smiles Dental Clinic, Mohali, Punjab, India; 5 Dentistry in Motion, North York, Ontario, Canada; 6 St. Joseph Healthcare Centre, Toronto, Canada

**Keywords:** renal cell cancer, metastasis, oral, soft tissues

## Abstract

**Background** Renal cancer metastasis to oral region is very rare. Studies have been published analyzing the cases of metastatic tumors to the oral cavity by many researchers. Very few research studies have been conducted till date to analyze the renal cancer metastasis as the sole primary source to the oral soft tissues. The goal of this study was to examine the published cases of oral soft tissue metastasis from renal cell carcinoma as the only primary source from 1911 to 2022.

**Materials and Methods** An electronic search of the published literature was performed without publication year limitation in PubMed/Medline, Scopus, Google Scholar, Web of Science, Science Direct, Embase, and Research Gate databases, using mesh keywords like (“Renal cancer,” *or* “Renal carcinoma” *or* “Renal cell cancer” *or* “Renal cell carcinoma”), *and* (“Metastasis” *or* “Metastases”), *and* (“Oral soft tissues” *or* “Tongue” *or* “Palate” *or* “Tonsil” *or* “Buccal mucosa” *or* “Salivary glands”). We also searched related journals manually and the reference lists.

**Results** Our research revealed a total of 226 relevant articles with 250 patients. Parotid glands and tongue were the most common sites of metastasis. 23% patients died with a survival time of 10 days to 4 years.

**Conclusions** Oral soft tissue metastasis from renal cell carcinoma has a bad prognosis. More cases need to be published in order to raise awareness of these lesions.

## Introduction

According to GLOBOCAN databases, renal cell carcinoma (RCC) is one of the lethal neoplasms leading to approximately 2% of global cancer diagnoses and deaths, projecting to increase in burden worldwide. 1 RCC is the most common primary renal neoplasm constituting about 80 to 85% of all renal malignancies. Renal cortex and pelvis are the most predominant sites. In the recent years, the incidence of RCC has increased worldwide owing to the development of newer imaging aids. In most of the cases, RCC is diagnosed as an incidental finding during radiological investigations. Only in 10% of patients, “classic triad” of symptoms (i.e., hematuria, flank pain, and palpable masses) has been noticed. 2 One of the unique features of RCC is its long-term asymptomatic clinical behavior and high risk of distant organ metastasis in the advanced stages. Studies have reported that approximately 18% of patients with RCC have metastasis at the time of diagnosis, and in more than 50% of cases, metastasis is detected during the follow-up period after nephrectomy. 3 The most common organs involved in distant metastasis of RCC are lungs (45%), followed by bones (30%), lymph nodes (22%), liver (20%), adrenal glands (9%), and brain (9%). 4 Metastasis from RCC to oral cavity is very rare—tongue, gingiva, and mandible being the most affected sites. 5 The prognosis of metastatic lesions in the oral cavity is unfavorable because of their late detection owing to resemblance of benign growths. Literature has reported several studies analyzing the metastatic tumors to the oral region. 6 7 8 But a very few research work has been published till date to analyze solely the RCC metastasis to the oral soft tissues (OSTs). Thus, this review was conducted to examine the published cases of OST metastasis (OSTM) from RCC as the sole primary source in the literature from 1911 to 2022, and to learn about their characteristics.

## Materials and Methods

The current research was carried out following the guidelines of Preferred Reporting Items for Systematic Reviews and Meta-Analyses. Owing to the nature of the current review, any ethical approval was not required.

### Focused Question

To conduct the study, CoCoPop (context, condition, population) framework, designed by Joanna Briggs Institute, was used focusing on the research question “how many cases of RCC metastasizing to OST have been documented in the literature, and what is the prognosis of these metastatic lesions.”

Pop (population): Patients with RCC.
Co (condition): Salivary gland metastasis.
Co (context): Characteristics of these patients.

### Search Strategy for Identification of Studies

**Fig. 1:**
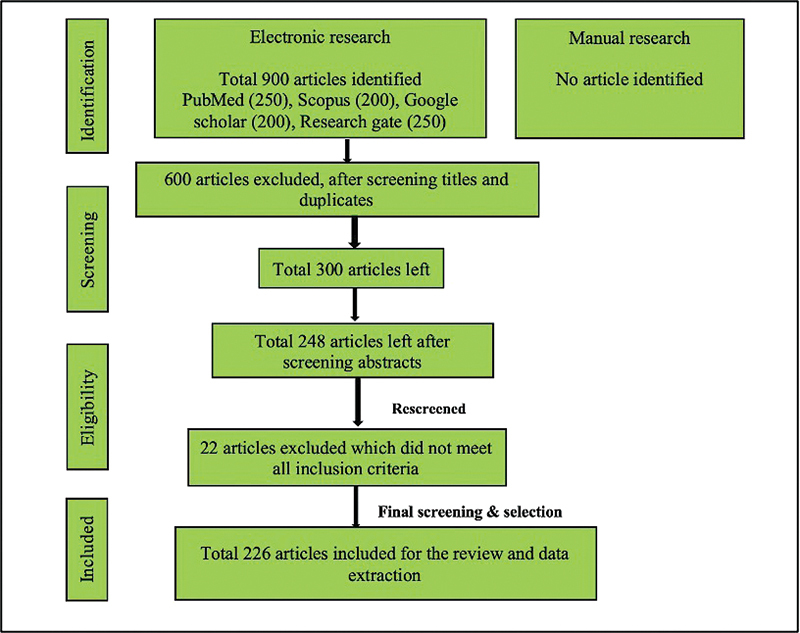
PRISMA flowchart showing search strategy.

An electronic search of the published literature was performed without publication year limitation in PubMed/Medline, Scopus, Google Scholar, Web of Science, Science Direct, Embase, and Research Gate databases, using mesh keywords such as “Renal cancer” *or* “Renal carcinoma” *or* “Renal cell cancer” *or* “Renal cell carcinoma” *and* “Metastasis” *or* “Metastases,” *and* “Oral soft tissues” *or* “Tongue” *or* “Palate” *or* “Tonsil” *or* “Buccal mucosa” *or* “Salivary glands.” We also searched all related journals manually. The reference list of all articles was also checked ( Fig. 1 ).

### Screening of Studies

The current review involved three steps of screening the studies. In the first step, titles were reviewed by two authors (H.V.P., R.S.) independently and duplicates were removed. Then the other two authors (S.G., H.S.) reviewed the selected abstracts of all the reports independently. The reviewers were calibrated on the basis of their assessment of their titles and abstracts of the first 50 references retrieved. The kappa value of agreement between reviewers was 0.82. If the title/abstracts met the eligibility rule, they were included in the study. In the final stage, the text of selected studies was screened by the remaining two authors (J.K., J.K.) separately. The full report was collected, discussed, and resolved for cases among all authors that appeared to fit the inclusion criteria or for which evidence was insufficient to make a clear determination.

### Inclusion Criteria

- Confirmed cases of OSTM from RCC as the sole primary source were included. Articles included were from 1911 to 2022.
- *Type of studies* : Case reports, case series, retrospective analysis, clinicopathological studies, prospective studies, original researches, systematic reviews, and correspondence.
- Cases were selected beyond the restriction of limitations on parameters such as age, gender, ethnicity, or socioeconomic status.
- Articles published in any language were included.

### Exclusion Criteria

- Cases with no definite diagnosis of OSTM from RCC as the sole primary source.
- Publications reporting the OSTM from any site other than kidney.
- Cases with RCC metastasis to jaw bones were not included.
- Studies which did not provide individual patient's data were excluded.
- Review articles, editorials, conference abstracts, hypothesis articles, web news, media reports, animal studies.

### Outcome Measures

*Primary outcome measures* : To evaluate the number of cases of RCC metastasizing to OST documented in the literature.

*Secondary outcome measures* : To evaluate other factors such as world-wide distribution of cases of OSTM from RCC, patient's demographic details, associated risk factors, predominant site of OSTM, clinical features of these metastatic lesions, most prevalent type of metastatic RCC, and type of therapies used.

### Risk of Bias Assessment

Most of the studies included in this review were case reports and case series. Risk of bias was appraised following CARE and Strengthening the Reporting of Observational Studies in Epidemiology (STROBE) checklists. 9 10 In several articles, there was missing information regarding many parameters used for data extraction. We tried reaching the authors of those cases to clarify this bias; however, we were unable to recover the missing information.

### Data Extraction and Analysis

After study selection, screening, and a thorough examination, the data were extracted. The information gathered was cross-checked and tabulated into three tables ( Tables 1 2 3 ). In case of missing data, 6 weeks' time was given to gather the information. If the information was still missing, we then indicated the missing data as “Not available (NA)” in the text and in the tables. The results were expressed in descriptive statistics. The overall survival rate was calculated by survival analysis with Kaplan–Meier curves.

**Table 1:** Details of publications included in the current review (1911–2022)

Sl. no.	Authors	Year	Country	Type of study	Total no. of patients
1.	Kostenko	1911	Russia	CR	1
2.	Coenen	1914	Germany	CR	1
3.	Branch and Norton	1928	USA	CR	1
4.	McNattin and Dean Jr	1931	USA	CR	1
5.	Trinca and Willis	1936	USA	CR	1
6.	Salman and Darlington	1944	USA	OR	1
7.	Scharg and Jordan	1945	USA	CR	1
8.	Bernier and Tiecke	1951	USA	CR	1
9.	Salman and Lengel	1954	USA	OR	1
10.	Persson and Wallenius	1961	Europe	CR	1
11.	Patey et al	1965	UK	CP	1
12.	Cranin et al	1966	USA	CR	1
13.	Del Carmen et al	1970	USA	CR	1
14.	Lansigan et al	1973	USA	CR	1
15.	Satomi et al	1974	Japan	CS	3
16.	Morri	1975	Japan	CR	1
17.	Batres et al	1978	USA	CR	1
18.	Friedlander and Singer	1978	USA	CR	1
19.	Nagayama and Oka	1979	Japan	CR	1
20.	Susan et al	1979	USA	CR	2
21.	Buchner and Begleiter	1980	Israel	CR	1
22.	Kucan et al	1981	USA	CS	1
23.	Bucin et al	1982	Europe	CR	1
24.	Fitzgerald et al	1982	USA	CR	1
25.	Nishimura et al	1982	Japan	SR	1
26.	Percival and Curt	1982	UK	CR	1
27.	Schaffner et al	1982	USA	CS	1
28.	Sist et al	1982	USA	CR	1
29.	Fay and Weir	1983	USA	CR	1
30.	Bedrosian et al	1984	USA	CR	1
31.	Lutcavage et al	1984	USA	CR	1
32.	Smits and Slootweg	1984	Netherland	CR	1
33.	Zohar et al	1985	Israel	CR	1
34.	Hessen et al	1986	USA	CS	1
35.	Kitao et al	1986	Japan	CR	1
36.	Harrison et al	1987	UK	CR	1
37.	Inai et al	1987	Japan	CR	1
38.	Kapoor et al	1987	India	CR	1
39.	Matsumoto et al	1987	Japan	CR	1
40.	Som et al	1987	USA	RA	1
41.	Tsianos et al	1987	Greece	CR	1
42.	Madison and Frierson	1988	USA	CR	1
43.	Gunbay et al	1989	Turkey	CR	1
44.	Hagen et al	1989	Germany	CR	1
45.	Melnick et al	1989	USA	CR	1
46.	Muller Mattheis et al	1989	Germany	CR	1
47.	Owens et al	1989	USA	CS	2
48.	Coppa and Oszczakiewicz	1990	USA	CS	2
49.	Martinez-Conde et al	1990	Spain	CR	1
50.	Ord et al	1990	UK	CR	2
51.	Pisani et al	1990	Italy	CR	1
52.	Tsuta et al	1990	Japan	CR	1
53.	Ishikawa et al	1991	Japan	CR	1
54.	Pastermoli	1991	Italy	CR	1
55.	Sarangi and Hameed	1991	UK	CR	1
56.	Okabe et al	1992	Japan	CR	1
57.	Ravi et al	1992	India	CR	1
58.	Shibayama et al	1993	Japan	CR	1
59.	Corsi et al	1994	Italy	CS	1
60.	Ziyada et al	1994	UK	CR	1
61.	Airoldi et al	1995	Italy	CR	1
62.	Borghi et al	1995	Italy	CR	1
63.	Stanley et al	1995	UK	CR	2
64.	Sykes et al	1995	UK	CR	1
65.	Aguirre et al	1996	USA	CR	1
66.	Ficcara et al	1996	Italy	CR	1
67.	Green et al	1997	UK	CR	1
68.	Konya et al	1997	Japan	CR	1
69.	Gangopadhyay et al	1998	Saudi Arabia	CR	1
70.	Garcia Lozano et al	1998	Spain	CR	1
71.	Tomita et al	1998	Japan	CR	1
72.	Vara et al	1998	Spain	CR	1
73.	Adil et al	1999	Turkey	CR	1
74.	Navarro et al	2000	Spain	CS	2
75.	Kundu et al	2001	UK	CR	1
76.	Li et al	2001	Germany	CR	1
77.	Fukuda et al	2002	Japan	RA	1
78.	Ieva et al	2002	Italy	CR	1
79.	Mekni et al	2002	Africa	CR	1
80.	Park et al	2002	USA	RA	1
81.	Pritychyk et al	2002	USA	RA	2
82.	Goel et al	2003	UK	CR	1
83.	Lang et al	2003	Ireland	CR	1
84.	Gogus et al	2004	Turkey	CR	1
85.	Jayasooriya et al	2004	Sri Lanka	CR	1
86.	Kyan et al	2004	Japan	CR	1
87.	Marioni et al	2004	Italy	CR	1
88.	Lim et al	2005	Australia	CR	1
89.	Seijas et al	2005	Spain	CR	1
90.	Tachi et al	2005	Japan	CR	1
91.	Cochrane et al	2006	UK	CR	1
92.	Huang et al	2006	Taiwan	CS	1
93.	Moudouni et al	2006	France	CR	1
94.	Pomar Blanco et al	2006	Spain	RA	1
95.	Porter et al	2006	UK	CR	1
96.	Stanczyk et al	2006	Poland	CR	1
97.	Stodulski et al	2006	Poland	CR	1
98.	Torres-Carranza et al	2006	Spain	CR	1
99.	Andreades et al	2007	Greece	CR	1
100.	del Rosario Regaldo et al	2007	USA	CR	1
101.	Kondo et al	2007	Japan	CR	2
102.	Newton et al	2007	UK	CR	1
103.	Tanaka et al	2007	Japan	CR	1
104.	Azam et al	2008	UK	CR	1
105.	Kalpan et al	2008	Turkey	CR	1
106.	Longo et al	2008	Italy	CR	1
107.	Mrena et al	2008	Finland	SR	3
108.	Narena-Matamala et al	2008	Chile	CR	1
109.	Spreafico et al	2008	Italy	CR	1
110.	Will et al	2008	USA	CR	1
111.	Basely et al	2009	France	CR	1
112.	Choi et al	2009	Japan	CR	1
113.	Dahlstrom	2009	Australia	CR	1
114.	Kella et al	2009	USA	CR	1
115.	Laco et al	2009	Czech Republic	CR	1
116.	Lee et al	2009	Japan	CR	1
117.	Maestre-Rodríguez et al	2009	Spain	CR	1
118.	Makos et al	2009	Greece	CR	1
119.	Massaccesi et al	2009	Italy	CR	1
120.	Novak et al	2009	Europe	CR	1
121.	Altinel et al	2010	Turkey	CR	1
122.	Miah et al	2010	UK	CR	1
123.	Ogunyemi et al	2010	USA	RA	1
124.	Saghravanian et al	2010	Iran	CR	1
125.	Syrolo et al	2010	Poland	CR	1
126.	Wayne et al	2010	USA	CR	1
127.	Zhang et al	2010	China	CR	1
128.	Eivazi Ziaei et al	2011	Iran	CR	1
129.	Ito et al	2011	Japan	CR	1
130.	Morvan et al	2011	France	CR	1
131.	Sinha et al	2011	India	CR	1
132.	Wadasadawala et al	2011	India	CR	1
133.	Yoshitomy et al	2011	Japan	CR	1
134.	Balliram et al	2012	Spain	CR	1
135.	Deeb et al	2012	USA	CR	1
136.	Ganini et al	2012	Italy	CR	1
137.	Ghazali et al	2012	UK	CR	1
138.	Gi-Julio et al	2012	Spain	CR	1
139.	Lau et al	2012	Australia	CR	1
140.	Lawlor et al	2012	USA	CR	1
141.	Novák et al	2012	Europe	CR	1
142.	Schwab and Lee	2012	USA	CR	1
143.	Serouya et al	2012	USA	CR	1
144.	Marcotullio et al	2013	Italy	CR	1
145.	Mazeron et al	2013	France	CR	1
146.	Ray et al	2013	India	Co	1
147.	Sikka et al	2013	India	Co	1
148.	Vegara et al	2013	Spain	CR	1
149.	Yanlan et al	2013	China	CR	1
150.	Abbaszadeh Bidokhty et al	2014	Iran	CR	1
151.	Akatiken et al	2014	Turkey	CR	1
152.	Hosan-Centenero et al	2014	Spain	CR	1
153.	Khobragade et al	2014	India	CR	1
154.	Kotak and Merrick	2014	UK	CR	1
155.	Kumar et al	2014	India	OR	1
156.	Kwak et al	2014	South Korea	CR	1
157.	Maralani et al	2014	Canada	CR	1
158.	Milner et al	2014	Poland	CR	1
159.	Suojanen et al	2014	Finland	CR	1
160.	Tunio et al	2014	Saudi Arabia	CR	1
161.	Udagar and Rungta	2014	USA	CR	1
162.	Altuntas et al	2015	Turkey	CR	1
163.	Bulguru et al	2015	Turkey	CR	1
164.	Jatti et al	2015	India	CR	1
165.	Kolokythas et al	2015	USA	CR	1
166.	Mellioni et al	2015	Italy	CR	1
167.	Piggati et al	2015	Brazil	CR	1
168.	Shi et al	2015	China	CR	1
169.	Ali et al	2016	Sudan	CR	1
170.	Balaban et al	2016	Turkey	CR	1
171.	Berkiten et al	2016	Turkey	CR	1
172.	Guimaraes et al	2016	Brazil	CR	1
173.	Hussain et al	2016	USA	CR	1
174.	Kudva et al	2016	India	CR	1
175.	Majewska et al	2016	Poland	CP	9
176.	Masaharu et al	2016	Japan	CR	1
177.	Renda et al	2016	Turkey	CR	1
178.	Selvi et al	2016	Turkey	CR	1
179.	Shirazian and Bahrami	2016	Iran	CR	1
180.	Wang et al	2016	Japan	CR	1
181.	Erkilic et al	2017	Turkey	CR	1
182.	Georgy et al	2017	India	CR	1
183.	Leider et al	2017	Germany	RA	2
184.	Nifosi et al	2017	Spain	CR	1
185.	Raiss et al	2017	Morocco	CR	1
186.	Rocca et al	2017	Italy	CR	1
187.	Danic et al	2018	Europe	CP	1
188.	Franzen et al	2018	Germany	CR	2
189.	Gandala et al	2018	India	CR	1
190.	Higuera et al	2018	Argentina	CR	1
191.	Khde et al	2018	USA	CR	1
192.	Kishore et al	2018	India	CR	1
193.	Morita et al	2018	Japan	CR	1
194.	Sahin et al	2018	Turkey	RA	1
195.	Vasilyeva et al	2018	USA	CR	1
196.	Abro et al	2019	USA	CR	1
197.	Boulanger et al	2019	France	CR	1
198.	Kilickap et al	2019	Turkey	CR	1
199.	Kizaekka et al	2019	UK	CR	1
200.	Nesbitt et al	2019	Australia	CR	1
201.	Netto et al	2019	Brazil	CR	1
202.	Sydney et al	2019	Turkey	CR	1
203.	Albsoul et al	2020	Jordan	CR	1
204.	Fejsa-Levakov et al	2020	Europe	CR	1
205.	Halnony et al	2020	Turkey	CR	1
206.	Kovalevsky et al	2020	Brazil	CR	1
207.	Nisi et al	2020	Italy	CR	2
208.	Patel et al	2020	USA	CR	1
209.	Stojanović et al	2020	Europe	CR	1
210.	Tsitsika et al	2020	Greece	CR	1
211.	Vuckovic et al	2020	Podgorica	CR	1
212.	Cecen et al	2021	Turkey	CR	1
213.	Chelliah et al	2021	USA	CR	1
214.	Darshan et al	2021	India	CR	1
215.	Gopan et al	2021	India	CR	1
216.	Martire et al	2021	Brazil	CR	1
217.	Santana et al	2021	Brazil	CR	1
218.	Torchalla et al	2021	Poland	CR	2
219.	Villanueva et al	2021	Spain	CR	1
220.	Williams et al	2021	USA	CR	1
221.	Baez et al	2022	USA	CR	1
222.	Krawczyk et al	2022	Poland	CR	1
223.	Lavanya et al	2022	India	CR	1
224.	Parosanu et al	2022	Romania	CR	1
225.	Singla et al	2022	India	PS	1
226.	Wallace et al	2022	UK	CR	1

Abbreviations: CR, case report, Co, correspondence, CP, clinicopathological study, CS, case series, OR, original research, PS, prospective study, RA, retrospective analysis, SR, systematic review, UK, United Kingdom, USA, United States of America.

**Table 2:** Clinical details of patients with renal cell carcinoma mediatizing to oral soft tissues (1911–2022)

Pt. no.	Age (in years)	Gender	Previous history of RCC/side of kidney	Medical history	Chief complaint	Oral site of metastasis	Clinical findings	Provisional diagnosis	Oral soft tissue as the initial site of metastasis?	Time of diagnosis of metastasis after nephrectomy	Any other organs involved in metastasis	Final diagnosis of metastatic RCC
1.	43	M	NA	NA	NA	T (SNA)	NA	NA	NA	NA	NA	CCC
2.	62	F	NA	NA	NA	T (SNA)	NA	NA	NA	NA	NA	CCC
3.	64	M	N	N	Difficulty in swallowing for 2 y	G (mand, R, ant)	Small epulis like growth	Epulis, SCC	Y	–	NA	CCC
4.	58	M	NA	NA	NA	T (SNA)	NA	NA	NA	NA	Lung, heart, skin	CCC
5.	57	M	NA	NA	NA	T (SNA)	NA	NA	NA	NA	NA	CCC
6.	54	M	NA	NA	NA	HP	NA	NA	NA	NA	NA	CCC
7.	61	M	NA	NA	NA	T (SNA)	NA	NA	NA	NA	Lung	CCC
8.	47	M	Y/NA	NA	NA	LL	NA	NA	N	9 mo	Lung, scalp	CCC
9.	62	M	NA	NA	NA	G (SNA)	NA	NA	NA	NA	Lung, bone, scalp	CCC
10.	60	M	NA	NA	NA	G (mand)	NA	NA	NA	NA	N	CCC
11.	63	F	N	NA	Pulsatile mass for 1 y	P (SNA)	Soft, fluctuant swelling	SGT	Y	–	NA	CCC
12.	NA	M	NA	NA	NA	G (mand)	NA	NA	NA	NA	NA	CCC
13.	77	M	Y/L	N	Painful mass	T (SNA)	Firm, painful swelling	SCC	N	3 wk	NA	CCC
14.	74	M	NA	NA	Difficulty in swallowing	Uvula	Soft, vascular mass	SCC	NA	–	NA	NA
15.	41	F	Y/L	NA	Abnormal sensation	T (SNA)	Small, soft swelling	PG	N	4 mo	Lung, LN, R kidney	CCC
16.	66	M	Y/L	NA	Difficulty in eating	P (SNA)	Firm, fixed mass	SGT	N	1 y	Bone	CCC
17.	69	M	Y/L	NA	Painless growth for few months	G (max R, post)	Soft, painless swelling, bleed on touch	PG, PGCG	N	2 y 3 mo	Brain, LN	CCC
18.	NA	NA	Y/R	Appendicitis	Difficulty in eating	BM (SNA)	Pedunculated, thumb-tip-sized, polyp-like tumor	NA	N	6 y	N	CCC
19.	63	M	NA	NA	Painless mass	Chin	Pedunculated crimson nodule	PG	NA	NA	NA	CCC
20.	84	M	Y/NA	NA	Swelling	T (tip)	Soft, painful swelling	SCC	N	NA	Lung	CCC
21.	43	F	Y/L	N	Painful swelling	HP	Polyp-like swelling developing into sequestrum	NA	N	15 y	N	CCC
22.	53	M	NA	NA	NA	HP	NA	NA	NA	NA	Lung, bone, liver	CCC
23.	62	M	NA	NA	NA	HP	NA	NA	NA	NA	N	CCC
24.	NA	NA	Y/NA	NA	Painful swelling	G (SNA)	Soft swelling	PG, PGCG	N	NA	NA	CCC
25.	55	M	N	N	Intraoral mass × 3–4 mo	P (R)	Soft ass	SGT	Y	–	NA	CCC
26.	65	M	Y/NA	N	Multiple swellings	G (SNA), P (SNA)	Nodular masses	PGCG, SGT	N	6.5 y	NA	CCC
27.	63	M	Y/NA	N	Difficulty in eating	G (SNA), T (dorsum)	Soft, vascular swelling on gingiva; soft pulsatile mass on tongue	PG	N	NA	Brain	CCC
28.	72	M	NA	NA	NA	G (SNA)	NA	NA	NA	NA	NA	CCC
29.	71	F	N	N	Mass below right angle of mandible for 9 mo	P (R)	Pulsatile, mobile swelling	SGT	Y	–	Liver, lung	CCC
30.	64	M	NA	NA	NA	G (SNA)	NA	NA	NA	NA	NA	CCC
31.	62	M	N	N	Mass on left preauricular region	P (L)	Swelling	SGT	Y	–	NA	CCC
32.	18	F	Y/NA	NA	Swelling	G (SNA)	Soft swelling	PGCG	N	14 y	Multiple	CCC
33.	61	M	N	N	Painless mass in mouth	SMG (L)	Firm, non-tender swelling	SGT	Y	–	N	CCC
34.	55	M	NA	NA	Difficulty in eating	HP	Highly vascular mass	NA	NA	NA	NA	CCC
35.	60	F	Y/NA	NA	Painful mass on right preauricular region	P (R)	Firm, non-tender swelling	SGT	N	8.5 y	SMG	NA
36.	54	F	NA	NA	Gum mass	G (SNA)	Soft, erythematous swelling	PGCG	NA	NA	N	CCC
37.	52	M	N	N	Mass on left preauricular region for 2 mo	P (L)	Soft, fluctuant swelling	SGT	Y	–	Lungs, ribs, lumbar spine, and brain	CCC
38.	57	M	N	N	Mass on tongue	T (base)	Soft, vascular, mass	SCC, any oral malignancy	Y	–	Lung, bone	CCC
39.	64	F	Y/NA	N	Mass on right preauricular region for 2 mo	P (R)	Swelling	SGT	N	10 y	N	CCC
40.	42	M	Y/L	N	Bleeding and pain	T (left)	Swelling, bleed on touch	PG	N	2 y	Lung, bone	CCC
41.	70	M	N	N	Mass for few months	T (SNA)	Painless swelling	NA	Y	–	NA	CCC
42.	77	F	N	NA	Swelling	T (left)	Painless swelling	NA	Y	–	Lung	CCC
43.	42	NA	N	NA	Rapidly growing mass	P (SNA)	Soft, firm swelling	NA	Y	–	Thyroid, max sinus	NA
44.	78	M	NA	NA	Masses in oral cavity	G (max, mand)	NA	NA	NA	NA	Lung, brain	CCC
45.	58	M	NA	NA	NA	T (SNA)	NA	NA	NA	NA	Lung, liver	CCC
46.	60	M	NA	NA	Painful mass	P (L)	Painful swelling	SGT	N	NA	NA	CCC
47.	46	F	NA	NA	NA	G (SNA)	NA	NA	NA	NA	Bone	CCC
48.	72	M	NA	NA	Mass on left preauricular lesion for 2.5 y	P (L)	Swelling	SGT	Y	–	Liver, lungs, mediastinum, adrenal	NA
49.	47	F	NA	NA	NA	NA	NA	NA	NA	NA	Bone	CCC
50.	55	M	N	NA	Intraoral ass × 3 mo	P (R)	Swelling	SGT	Y	–	Chest, brain, bone	CCC
51.	75	F	Y/L	NA	Pulsatile mass for 10 wk	P (L)	Swelling	SGT	N	8 y	Recurrent renal disease	CCC
52.	42	M	N	NA	Pulsatile mass	P (L)	Soft fluctuant, tender swelling	SGT	Y	–	Perirenal LN	CCC
53.	55	M	Y/R	NA	Painful mass for 6 wk	P (R)	Soft swelling	SGT	N	7 y	Lungs, axillary lymph nodes	CCC
54.	NA	NA	NA	NA	NA	G (SNA)	NA	NA	NA	NA	NA	CCC
55.	58	M	NA	NA	NA	G (SNA)	Soft erythematous mass	NA	NA	NA	Brain, lung, biceps	CCC
56.	73	M	NA	NA	NA	G (SNA)	Erythematous, bleed on touch	NA	NA	NA	Brain	CCC
57.	59	M	N	N	Mass on left preauricular region for 2 mo	P (L)	Firm swelling	SGT	Y	–	Cerebellum, vertebrae	CCC
58.	51	M	Y/L	N	Swelling, facial weakness	P (R)	Firm swelling	SGT	N	5 y	NA	CCC
59.	59	F	Y/L	N	Growth	T (left border)	Firm swelling, indurated borders	NA	N	5 y	Lung, brain, liver, kidney	CCC
60.	62	M	N	N	Swelling	G (both max, and max)	Vascular mass	PG	Y	–	Lung	CCC
61.	71	M	Y/NA	NA	Mass on the left side of the mouth for 3 mo	P (L)	Firm swelling	SGT	N	4 mo	Radius	CCC
62.	58	M	NA	NA	Swelling	T (SNA)	Soft painful swelling	PG	NA	NA	Lung Brain	CCC
63.	55	F	Y/NA	NA	Bilateral masses for 3 mo	P (BL)	Painful swelling	SGT	N	7 y	NA	CCC
64.	41	M	Y/R	N	Difficulty in eating	T (base)	Pain, bleeding	NA	N	3 y	Lung, bone, lymph node	CCC
65.	44	M	Y/L	N	Pain in mouth	Cheek, UL	NA	NA	N (46, 51 mo)	4 y	NA	CCC
66.	59	M	N	N	Swelling	T (base)	NA	NA	Y	–	N	CCC
67.	51	M	NA	NA	Swelling	T (left surface)	Soft mass	PG	NA	NA	Lung, liver, brain	NA
68.	63	M	N	NA	Mass for 1 yr	P (R)	Soft mass	SGT	Y	–	Liver, pancreas	CCC
69.	59	M	N	NA	Mass for 3 wk on the left side of the mouth	P (L)	Soft mass	SGT	Y	–	Perirenal lymph nodes	CCC
70.	40	M	N	NA	Swelling	P (R)	Soft mass	SGT	Y	–	NA	CCC
71.	59	M	N	NA	Swelling	P (R)	Soft mass	SGT	Y	–	NA	CCC
72.	82	F	N	N	Gradually increasing swelling	T (Tip)	Pedunculated, reddish blue, hemorrhagic lesion	PG, Primary tongue carcinoma, Metastatic	Y	–	Brain	CCC
73.	73	F	NA	NA	Difficulty in swallowing	Wharton duct (R)	NA	NA	NA	NA	NA	CCC
74.	NA	NA	NA	NA	Difficulty in swallowing	To	Soft fluctuant swelling	Peritonsillar abscess	NA	NA	NA	CCC
75.	59	M	N	N	Tumor for 2 y	T (SNA)	Soft fluctuant swelling	PG, Primary or metastatic tongue carcinoma	Y	–	LN	CCC
76.	48	M	N	NA	Mass on left side neck for 3 mo	P (L)	Soft, painless mass	SGT	Y	–	Right adrenal	CCC
77.	56	F	Y/ L	N	Painful mass	To (R)	Soft, fluctuated swelling	NA	N	6 mo	NA	CCC
78.	52	M	Y/L	N	Rapidly growing mass on tongue	T (left surface)	Pedunculated, reddish blue, hemorrhagic lesion	PG, Primary or metastatic tongue carcinoma	N	10 mo	Lung, brain, skin	CCC
79.	50	M	Y/R	N	Rapidly increasing painful mass for 2 mo	P (L)	Enlarged parotids, a solid, well-circumscribed tumor, deeply adhering without FN involvement	SGT	N	5 y	N	CCC
80.	52	M	Y/R	NA	Intraoral mass	P (R)	Rapidly increasing painless mass	SGT	N	5 mo	LN	CCC
81.	50	M	N	NA	Mass	T (SNA)	Soft swelling	PG	Y	–	Lung	CCC
82.	NA	NA	N	NA	Swelling	T (SNA)	Firm swelling	SCC, PG	Y	–	N	CCC
83.	61	M	N	N	Facial weakness on right side	Face Outer skin (R)	NA	NA	Y	–	R. adrenal, bone, skin, pulmonary, cerebral	CCC
84.	63	M	Y/NA	NA	Rapidly growing masses on both sides of face	P (BL)	3 × 2.5 cm firm mass in both glands	SGT	N	14 y	NA	CCC
85.	74	M	NA	NA	Swelling on tongue	T (SNA)	NA	NA	NA	NA	NA	CCC
86.	83	F	Y/NA	NA	Mass ×2 mo on the left preauricular region	P (L)	Firm, nodular swelling	SGT	N	10 y	N	CCC
87.	63	M	Y/NA	NA	Swelling and pain on tongue	T (SNA)	Swelling firm	PG	N	20 y	NA	CCC
88.	83	F	Y/NA	NA	Mass ×2 mo on left preauricular region	P (L)	Firm, nodular swelling	SGT	N	10 y	Y	CCC
89.	60	M	NA	NA	Dysphagia	T (SNA)	NA	NA	NA	NA	Lung	CCC
90.	70	M	NA	NA	Swelling	LL	NA	NA	NA	NA	Abdomen	CCC
91.	62	M	NA	NA	Swelling on tongue	T (base)	Soft sessile mass	PG	NA	NA	Lung	CCC
92.	45	M	Y / L	N	Dyspnea, cough, lethargy for 2 mo	T (tip)	Pedunculated, firm mass	NA	N	2 mo	Lung, nose	CCC
93.	59	F	Y/L	NA	Swelling on the left side of the face	P (L)	Firm, nodular	SGT	N	10 y	Other kidney	CCC
94.	57	F	N	N	Painful swellings on the left side of the face for 3 wk	BM (L)	3.3-cm oval shaped, hard swelling	NA	Y	–	Humerus	CCC
95.	66	M	Y/L	N	Discomfort in mouth	T (base)	Tumor mass	NA	N	3 y	Lung	CCC
96.	87	F	Y/R	N	Exophytic ulcerated mass	T (post)	Malignant oral tumor	NA	N	10 y	Lung, liver, pancreas, thyroid	CCC
97.	86	M	Y/L	NA	Multiple masses in oral cavity, face, neck, and scalp	Lip, SP	Popular masses	NA	N	4 y	Lung, leg, bone	CCC
98.	67	M	N	N	Mass on the left side of the mouth for 4 mo	P (L)		SGT	Y	–	Adrenals, lung, LN	CCC
99.	73	M	Y/L	N	Tumor	T (left side)	Partially ulcerated protruding mass	PG	N	10 y	Scapula, choroid, LN, brain	CCC
100.	41	M	NA	NA	Swelling on tongue and scalp	T (SNA)	Soft mass	NA	NA	NA	Lung, scalp, bone, brain	CCC
101.	76	F	Y/R	NA	Hemoptysis, dysphagia	T (base)	Fungating, bluish-purple lesion without cervical lymphadenopathy	NA	N	8 y	Lung, liver	CCC
102.	83	M	Y/NA	N	Swelling	SMG (SNA)	Firm, nodular lesion	SGT	N	10 y	N	CCC
103.	NA	NA	NA	NA	NA	P (SNA)	NA	NA	NA	NA	NA	CCC
104.	36	M	N	N	Rapidly growing lesion, abscess in the chin	Chin	Afebrile, erythematous, tender lesion	Abscess	Y	–	N	CCC
105.	61	M	Y/NA	NA	Difficulty in swallowing	To (R)	Pedunculated mass	NA	N	11 mo	Lung, brain	CCC
106.	49	F	NA	NA	Difficulty in breathing	T (SNA)	Smooth, painless swelling	Primary oral tumor	NA	NA	Lung	CCC
107.	NA	NA	Y/L	NA	Rapidly growing mass	P (SNA)	NA	NA	N	5 y	NA	CCC
108.	NA	NA	Y/L	NA	Growth on left side	P (SNA)	NA	NA	N	10 y	NA	CCC
109.	81	M	Y/R	Hypothyroidism, BPA	Tumor like mass for weeks	T (Post lat)	Polylobate fragile mass 2 × 1 cm	Oral CCC	N	3 y	Lung	CCC
110.	74	M	Y/N	N	Painful swelling on the right side	SMG (R)	Firm mass	NA	N	NA	NA	CCC
111.	78	M	Y/NA	N	Painful swelling on the right side	SMG (R)	Firm mass, non-fixed	NA	N	NA	NA	CCC
112.	74	F	Y/R	N	Right side preauricular swelling for 3 mo	P (R)	Painful, firm, 2 × 2 cm, no FN involvement	SGT	N	7 y	Ad gland	CCC
113.	82	m	Y/L	N	Painless mass	T (right side)	Soft elevated smooth red mass no bleeding	Na	N	4 y	Lung brain	CCC
114.	78	M	N	S	Difficulty in swallowing for 6 wk	T (R side ant 2nd/3rd)	Pedunculated mass	SCC	Y	–	N	CCC
115.	68	M	N	S	Gradually increasing painless mass in right periauricular region for 1 y	P (R)	Hard, firm, nontender, deeply adhering mass	SGT	Y	–	Lung, liver	CCC
116.	68	M	NA	NA	Swelling on tongue	T (SNA)	Soft swelling	PG	NA	NA	NA	CCC
117.	58	F	N	NA	Tender mass	P (R)	Painful swelling	SGT	Y	–	Meatus	CCC
118.	75	F	Y/NA	NA	Painful mass	P (L)	Tender mass	SGT	N	9 y	Contralateral kidney, lung, bone	CCC
119.	62	M	YNA	NA	Painful mass for few years	P (L)	Tender mass	SGT	N	5 y	Y	CCC
120.	74	M	N	Polyarthralgia	Swelling in lower gingiva for 1 mo	G (mand, A, BL)	Painless, erythematous, vascular, granulomatous lesion of 3 × 2 cm	NA	Y	–	Lung, brain	NA
121.	67	M	Y/R	CRF	Swelling	P (R)	Deeply adherent painless mass	NA	N	15 y	LN	CCC
122.	63	M	Y/R	N	Pain in right neck and tongue	T (SNA)	Swelling attached to FOM	NA	N	4 mo	LN	CCC
123.	46	F	Y/NA	N	Swelling on tongue	T (SNA)	Soft nontender mass	PG, OSTT	N	3 y	Lymph node	CCC
124.	64	M	Y/L	N	Swelling right auricle	P (R)	Round, painless, immobilized mass, 2 × 2 cm	SGT	N	10 y	N	CCC
125.	70	M	Y/R	NA	Lump on tongue	T (ant, right)	Nodule	Oral metastatic tumor	N	18 y	Multiple	CCC
126.	67	F	N	NA	Swelling on tongue while biting	T (ant 3rd)	Soft, vascular, painful nodule	NA	Y	–	Thyroid	CCC
127.	75	M	N	N	Swelling in right preauricular region for 6 mo	P (R)	Firm nodular mass, 4 × 3 mm	Warthin tumor	Y	–	Liver, vertebrae, lung, adrenals	CCC
128.	78	M	Y/R	Cholecystectomy	Rapidly increasing swelling for 2 mo	P (SNA)	Hard, painless mass of 3 × 3 cm	SGT	N	3 y	N	CCC
129.	52	M	N	N	Gingival bleeding, swelling	G (max, ant)	Polypoid mass	PG	Y	–	Phalanx of finger	CCC
130.	63	M	NA	NA	Gum mass	G (max)	Exophytic growth	Epulis	NA	NA	Femur, vertebrae, ribs, pelvis	CCC
131.	76	M	Y/L	S, HT, COPD, BA, anemia	Difficulty in eating	To (R)	Grey, vegetated mass	NA	N	6 y	Lung, bone	CCC
132.	63	F	Y/R	N	Swelling	T (right side of mid of base)	Soft, smooth swelling	Lipoma, neuroma,	N	7 y	NA	CCC
133.	67	M	N	N	Rapidly growing swelling tongue lesion	T (dorsum)	Large, irregular, fungating, reddish-blue, 4.8 cm	SCC	Y	–	Lung, abdomen, throat	CCC
134.	61	F	Y/L	Mastectomy, BC	Painless and palpable swellings in thyroid and mid mouth region	SMG (R)	Firm mass	Thyroid tumor	N	7 y	Thyroid	CCC
135.	27	M	N	NA	Swelling on tongue	T (SNA)	Soft cystic swelling	NA	Y	–	Spine, lung, liver	CCC
136.	75	M	N	N	Swelling in upper front region	G (max, ant)	NA	NA	Y	–	Brain, lung	CCC
137.	59	M	N	N	Nodule on upper lip for 2 mo	UL	NA	NA	Y	–	Lung, brain	CCC
138.	61	F	N	N	Mass on the left side of the face	P (L)	Enlarged size of parotid region	SGT	Y	–	Skin, pancreas	CCC
139.	74	M	Y/L	N	NA	G (SNA)	NA	NA	N	1 y	Brain, lung, liver	TCC Renal pelvis
140.	47	M	Y/L	N	Swelling and severe pain in the upper gingiva	G (max)	Painful swelling	NA	N	8 mo	Lung	CCC
141.	60	M	Y/R	N	Swelling on the right side of mouth	P (R)	Painful swelling	SGT	N	3 y	N	CCC
142.	48	F	Y/R	N	Painful swelling	T (left lateral border)	Nodule crossing midline	OSTT	N	4 y	Bone, lymph node	CCC
143.	35	M	N	Abdominal lump	Gradually increasing swelling	P (L)	Firm swelling	SGT	Y	–	N	CCC
144.	48	M	Y/L	MM	Dysphagia and dysphonia and mass on the tongue	T (base)	(5 × 5 cm) exophytic swelling in the oropharynx with epicenter in the right base of tongue involving the tonsillar fossa and adjacent soft palate on right side, crossing midline	NA	N	5 y	Lung, bone, adrenal, lymph node	CCC
145.	47	M	N	N	Swelling on tongue	T (dorsum)	Pedunculated firm swelling	PG, primary carcinoma of oral cavity	Y	–	N	CCC
146.	72	M	N	N	Rapidly increasing painless mass	T (right side)	Soft swelling	NA	Y	–	Lung	CCC
147.	82	M	Y/R	CLL, left adrenalectomy	Mass on R preauricular region for 18 mo	P (R)	Swelling	Lymphoma	N	19 y	N	CCC
148.	70	M	Y/R	N	Mass on tongue	T (left side)	Ulcerated, hemorrhagic mass	NA	N	15 y	Lung, bone, adrenal, lymph node	CCC
149.	64	F	Y/L	End-stage LC	Painless lump on tongue, growing rapidly in 4 wk	T (ant)	Firm, well-defined nontender lump, 5 cm	Metastatic	N	17 y	N	CCC
150.	65	M	Y/L	NA	Discomfort in left cheek	BM (L)	NA	NA	N	19 y	N	CCC
151.	79	F	Y/L	NA	Difficulty in eating	P (L)	Non tender soft swelling	SGT	N	16 y	N	CCC
152.	71	M	Y/R	HT, S	Mass for 2 y	P (R)	Non tender soft swelling without lymphadenopathy	NA	N	5 y	Pancreas	CCC
153.	63	F	Y/NA	NA	Painless, non-ulcerated, nodular mass	T (right side)	Swelling	PG	N	7 y	N	CCC
154.	63	M	NA	NA	Nodule	G (max, BL)	Exophytic mass	PGCG	NA	NA	MM	CCC
155.	NA	NA	Y/NA	NA	Hard mass	SMG (SNA)	NA	NA	N	9 y	NA	CCC
156.	72	F	Y/L	N	Hemoptysis, dysphagia	To (L)	Painful, exophytic, grayish, ulcerated mass	NA	N	3 y	Lung	CCC
157.	66	M	N	S, HT	Lesion on tongue	T (dorsum)	Exophytic mass	NA	Y	––	Visceral organs	CCC
158.	65	M	N	N	Growth for 2 mo	T (dorsum)	Pedunculated growth	NA	Y	–	Lung, muscles, LN	CCC
159.	73	M	N	Weight loss	Multiple painless swellings in the lower gingival region	G (mand, BL)	Three soft, reddish brown swellings (35–36, 37–38, 44–46 region) 3 × 2.5 cm, 2 × 1 cm, and 1 × 1 cm, respectively	NA	Y	–	N	CCC
160.	61	M	Y/R	Drug allergy, anemia, family history of RCC	Painless mass in right side of mouth	P (R)	Firm, painful mass	SGT	N	5 y	Lung, adrenal	CCC
161.	44	F	N	N	Painless mass in left side of mouth	P (L)	Painless swelling	SGT	Y	–	Lung, liver, bone	CCC
162.	80	M	Y/NA	HT	Swelling	T (dorsum)	Oval, reddish, sessile, painless mass	SGT, PG	N	4 y	N	CCC
163.	62	M	N	HT	Palpable swelling left preauricular	P (L)	Bony hard mass	PA	Y	–	N	CCC
164.	NA	NA	Y/NA	NA	Mass on left side	P (L)	Nodular, firm swelling	SGT	N	11 y	NA	CCC
165.	63	M	N	NA	Difficulty in eating for 3 mo	T (dorsum)	Soft swelling	Oral malignant tumor of clear cell variant	Y	–	Lung	CCC
166.	64	M	Y/NA	S, A	Rapidly growing swelling for 3 wk	LL	Soft erythematous, 4 cm	SCC, keratoacanthoma	N	6 mo	Lung	CCC
167.	62	M	Y/NA	N	Soft enlarging swelling	SP (R, post)	Red, fungating and ulcerated mass 3 × 2.5 cm. and bled on touch	NA	N	1 mo	N	CCC
168.	67	M	N	N	Rapidly growing mass in left side of mouth for 1 y	P (L)	Painless mass 2 × 2 cm	SGT	Y	–	N	CCC
169.	64	F	N	N	Slowly progressive left facial swelling for few months	P (BL), SMG (L)	Painless masses	SGT	Y	–	Thyroid	CCC
170.	67	M	N	HT, BA, prostate infection, UTI	Painful lesion for 3 mo	HP (R side)	Tender irregular lump ∼2–3 cm	NA	Y	–	N	CCC
171.	71	M	Y/R	N	Tumor mass on lower lip	LL	Soft, pulsatile mass 1.5 cm	NA	N	3 y	Lung, mediastinum	CCC
172.	70	M	Y/L	IHD, bypass surgery	Left cheek swelling, hematuria	P (L)	Hard, fixed tender mass of size 2 × 2 cm	SGT	N	15 y	Contralateral kidney, right adrenal	CCC
173.	64	M	Y/L	NA	Mass under his right ear for several weeks	P (R)	1.0 × 1.0 cm firm, painless, mobile mass	SGT	N	6 y	Lung	CCC
174.	67	M	N	N	Rapidly growing mass	T (dorsum)	Large, irregular, fungating, reddish-blue, 4.8-cm mass	SCC with clear cell variant	Y	–	Lung	CCC
175.	77	F	Y/BL	FNP (R side, hemi cranial pain (R side)	Right neck mass extending to right parotid and thyroid glands	P (R)	Hyper vascular mass, lymphadenopathy	Metastatic	N	3 y	Thyroid	CCC
176.	60	M	Y/L	A, tobacco	Asymptomatic rapidly increasing growth on the upper lip, present for 3 mo	UL	Exophytic, dome-shaped, ulcerated reddish pink nodule 2 × 1.5 cm	KA	N	5 mo	Lung, liver	CCC
177.	83	M	Y/R	N	Rapidly growing mass	P (R)	Firm swelling	SGT	N	10 yr	Cerebellum	CCC
178.	53	F	Y/L	Dialysis	Firm mass in the mouth	SMG (L)	Hard, round, fixed	SGT	N	4 yr	NA	CCC
179.	82	F	Y/L	NA	Rapidly enlarging mass	P (L)	Firm, nodular swelling	SGT	N	6 y	NA	CCC
180.	56	F	Y/R	Thyroid resection	Right preauricular painless mass present for 6 mo	P (R)	Smooth, firm, immobile and non-tender mass 3 × 3 cm	SGT	N	11 y	N	CCC
181.	60	M	N	N	Enlarging mass for 5 mo	G (mand, L, ant, post)	Painless, nodular fungating mass of 6 × 7 cm	Fibroma	Y	–	Lung	CCC
182.	66	F	Y/L	N	Painful swelling in the right side of mouth	P (R)	Well-defined, 37 × 21 mm in size, hypoechoic heterogeneous solid mass	NA	N	15 y	N	CCC
183.	70	M	Y/R	HT	Growing lesion in the right side of mouth	P (R)	Painless, soft, smooth 3 × 4 cm mass	NA	N	11 y	N	CCC
184.	31	F	Y/NA	NA	Gingival mass	G (mand)	NA	PGCG, PG	N	NA	Abdomen	CCC
185.	65	M	Y/R	SCC of right pinna	Right preauricular swelling	P (R)	Nodule	SGT	N	8 y	N	CCC
186.	36	F	N	AN	Nonhealing, painful ulcer in the right side for 2 mo	BM (R)	Ulcer proliferative growth 4 cm × 2 cm with raised shelf-like inferior margin and submucosal induration	Benign or malignant oral tumor	Y	–	Liver, bone	CCC
187.	66	F	N	NA	Painless, enlarging, hard, immobile mass, FNP	P (R)	Nontender, firm mass 5 × 4 cm, FNP	SGT	Y	–	N	CCC
188.	76	F	N	NA	Painless, enlarging, immobile mass	P (R)	Non-tender soft mass, 5 × 5 cm	SGT	Y	–	N	CCC
189.	97	F	N	NA	Hard, immobile mass	SMG (R)	Firm, nodular swelling	SGT	N	(At the time of diagnosis)	N	CCC
190.	68	M	N	NA	Growing firm painless mass for 4 mo	P (L)	Non-tender, firm swelling, 2.6 × 1.8 × 1.3 cm	SGT	Y	–	N	CCC
191.	69	M	N	NA	Palpable mass	P (L)	Firm hard swelling, 1.8 × 1.5 × 2 cm	SGT	Y	–	N	CCC
192.	NA	M	Y/NA	NA	Painless slowly growing mass for 3 mo	P (R)	Non-tender mass	SGT, metastatic	N	NA	Lung	CCC
193.	NA	F	Y/NA	NA	Tumor	Minor glands, left retromolar)	Soft, fluctuant swelling	SGT, metastatic	N	NA	Y	CCC
194.	NA	F	Y/NA	NA	Palpable tumor	P (R)	Firm, nodular mass, 1.5 cm	SGT	N	NA	Y	CCC
195.	60	F	N	NA	Swelling	P (R)	Multinodular palpable mass in the area of cicatrix	PA	Y		Y	CCC
196.	56	M	N	N	Swelling in upper region of mouth	HP	Firm, nodular swelling	Nodular fasciitis	Y	–	Ad, femur	CCC
197.	74	F	N	N	Swelling on left side of mouth	P (L)	Well-demarcated painless mass	SGT	Y	–	N	CCC
198.	51	M	Y/NA	NA	Swelling	G (max)	Soft, erythematous mass	PG	N	NA	Scalp, phalanx of the fifth digit	CCC
199.	45	M	N	Nephrolithiasis	Rapidly growing gingival mass	G (max, ant, BL)	Red-purple, sessile, exophytic mass extending to palate	Lymphoma, metastatic tumor	Y	–	N	CCC
200.	71	F	Y/NA	N	Tumor mass	T (tip)	Soft 10-mm swelling, bleed	PG	N	10 y	Hilar lymph node, lungs	CCC
201.	54	M	N	H/O extraction of 2nd molar 7 d before, hematuria	Oral lesion with erythema and swelling of the left lower side	G (mand, L, post)	Soft, erythematous mass	PG	Y	–	N	Collecting duct AC
202.	63	M	N	HT	Multiple painless reddish nodules on the gums, and the scalp	G (SNA)	3 in number, reddish in color, and intermittently bled ranging in size from 1 × 2 cm to 4 × 3 cm	PG, metastatic	Y	–	Lung, liver, bone	CCC
203.	56	NA	Y/NA	NA	NA	T (SNA)	NA	NA	N	5 mo	Lung, mediastinum	CCC
204.	74	NA	Y/NA	NA	NA	P (SNA)	NA	NA	N	6.5 y	N	CCC
205.	58	M	Y/NA	N	Growing mass, painful	G (max, R, ant)	Non-ulcerated mass without bleeding	Epulis	N	NA	Lung, brain, left occipital	CCC
206.	55	M	N	Tobacco, HT	Painful lesion	T (ant 2nd/3rd)	Exophytic growth	OSTT	Y	–	Lung, muscle	CCC
207.	NA	NA	NA	NA	Swelling on face	P (SNA)	NA	Oncocytoma	NA	NA	NA	CCC
208.	51	M	N	S, A	Difficulty swallowing, for 5 mo	T (base)	NA	NA	Y	–	Lung, liver, LN	CCC
209.	74	F	Y/L	Colorectal cancer	Rapidly growing painless mass in the left preauricular region	P (L)	Mobile mass	SGT/ Metastatic	N	11 y	Lung, liver	CCC
210.	80	F	Y/L	Colorectal cancer, parotid metastasis of left side	Rapidly growing painless mass in the right preauricular region	P (R)	Vascular mass 1.5 cm	Metastasis	N	17 y	N	CCC
211.	64	M	Y/L	Left choroid metastasis from RCC	Growth in the oral cavity for 1 mo	HP	Nodular mass, 5 × 3 cm, bleed easily	NA	N	1 y	Lungs, pancreas, adrenal. Infratemporal fossa	CCC
212.	74	F	Y/L	N	Hard nodule	SMG (R)	Firm vascular swelling	NA	N	11 y	N	CCC
213.	55	F	Y/R	N	Congestion	Uvula	Uvular erythematous mass with vascularity, ∼2 mm in size, and a 2-mm papillomatous lesion in the left hard palate	OSTT	N	3 y	Pancreas, max sinus, ethmoid sinus, lungs	CCC
214.	54	M	Y/L	N	Swelling over upper lip, scalp, and retromolar region	UL, retromolar (R)	Lip: Exophytic, firm, non-tender mass, 2 × 1.8 cm Retromolar: firm, diffuse growth 6 cm × 4.5 cm × 3.2 cm	PG, KS, AS	N	2 mo	Multiple	CCC
215.	75	M	Y/L	Myoepithelioma on left BM 7 y, ago	Rapidly growing lesion on left BM for weeks	BM (L)	Soft mass with a smooth surface 40 × 30 mm, facial asymmetry	Recurrent myoepithelioma	N	26 y	N	CCC
216.	61	M	Y/NA	Retroperitoneal lymph node dissection	Swelling tongue for 2 mo	T (tip)	Tender hemorrhagic mass	NA	N	20 y	N	CCC
217.	78	F	N	N	Enlarging soft tissue mass of several months	G (max, BL, ant)	Fluctuant, dark-red, exophytic lesion extending from the 12–21, 3.0 cm × 1.5 cm size	PG	Y	–	Right femoral head and greater trochanter	CCC
218.	54	M	Y/NA	COPD, DM, HT	Dyspnea	T (tip)	Sessile, papillary mass	Immune reaction	N	NA	Finger	CCC
219.	82	M	Y/NA	N	Mass on lower gingiva right side	G (mand, R, post)	Nodule with necrosis at center	PG, PGCG	N	NA	Femur, ribs	CCC
220.	63	M	Y/R	N	Difficulty in eating for 2 mo	To (R)	Exophytic, greyish and edematous mass	NA	N	5 y	N	CCC
221.	77	M	Y/L	N	Traumatic mass	T (dorsum)	Pedunculated, vascular lesion	PG, Benign	N	17 y	Liver, lung, atrium	CCC
222.	59	M	N	N	Painless ulcerative lesion in side of mouth	BM (L)	Non-fluctuant, ulcerated mass	SCC	Y	–	Lungs	CCC
223.	68	M	N	S, A, HT, gastritis, appendicitis	Rapidly increasing mass on the right side of the face	BM (R)	Firm, well-defined tumor-like lesion, slight elevation in the ipsilateral jugal mucosa	Benign or malignant soft tissue tumor	Y	–	N	CCC
224.	75	F	Y/L	S, A, recurrent RCC	Mass growing for 18 mo on left side of mouth	P (L)	Painless swelling, 4 × 4 cm	PA	N	10 y	N	CCC
225.	50	M	NA	NA	Rapidly growing mass on one side of face	P (SNA)	NA	NA	NA	NA	NA	CCC
226.	74	M	L	N	Difficulty in swallowing for several months	T (base)	Swelling	NA	N	2 y	Liver, thyroid, ileac bone	CCC
227.	50	M	N	N	Pain radiating to right ear for 5 mo	P (R)	Non tender swelling, trismus, lymphadenopathy	TN	Y	–	LN	CCC
228.	63	M	N	DM, HT	Rapidly growing intraoral mass for 2 mo	G (max, L)	Nodule, bleed easily, 5 × 3 cm, foul smell	PG, Malignant oral tumor	Y	–	Liver, lung, brain	CCC
229.	61	M	NA	NA	Rapidly increasing mass for 3 wk	T (left side)	Soft, erythematous swelling	PG, SCC	NA	NA	NA	CCC
230.	71	M	NA	NA	Swelling	BM (SNA)	Firm, nodular mass	Soft tissue tumor	NA	NA	NA	CCC
231.	59	F	N	Hip and back pain, GBS, right nephrectomy done for benign growth in 2014	Swelling on left side of mouth interfering dentures	BM (L)	Edentulous, with a pink-red, oval, ulcerated lesion measuring ∼38 × 25 × 17 mm	PG, SCC, Metastatic, soft tissue tumor	Y	–	Multiple sites	CCC
232.	53	M	N	Back pain	Rapidly growing tumor mass associated with bleeding, eating difficulty	G (mand, R, post)	Exophytic tumor mass, sized ∼ 6 × 3 cm, facial asymmetry	NA	Y	–	N	PCC
233.	65	F	Y/R	Appendectomy, cholelithiasis, and thyroid nodules, MM of RCC	Rapidly growing mass	BM (L)	Soft vascular mass with a smooth surface, 2.7 × 0.8 × 0.9 cm in growing 3rd molar region	Gingival hypertrophy	N	4 y	Adrenals, lung, spine, bone	CCC
234.	79	M	Y/L	N	Swelling	T (body)	Clearly demarcated smooth mass	NA	N	7 y	Lungs	CCC
235.	63	F	Y/L	TB	Rapidly growing mass for 3 wk	LL	2 × 1 cm hemorrhagic, ulcerated mass	NA	N	10 y	Brain, lung	CCC
236.	45	F	Y/NA	N	Painful bleeding nodule on chin for 4 mo	Chin	0.8 × 0.9 × 0.7 cm nodule	PG, KS,	N	7 y	N	CCC
237.	50	M	Y/R	NA	Swelling on upper lip	UP	Exophytic, firm, tender, pedunculated base, non-pulsatile, 4 × 3 cm	PG	N	3 y	N	CCC
238.	NA	NA	NA	NA	NA	P (SNA)	NA	NA	NA	NA	NA	CCC
239.	69	M	N	S, A	Nodule in the right parotid region for 3 mo	P (R)	Nodule, vascular	SGT	Y	–	N	CCC
240.	72	M	Y/R	N	Facial swelling for 1 y	P (SNA)	Nodular growth	SGT	N	8 y	N	CCC
241.	81	M	Y/L	HT, glaucoma, osteoarthritis	Tumor mass on left side of mouth	SMG (L)	Vascularized, solid mass, 24 × 21 × 26 mm in diameter	SGT	N	8 y	N	CCC
242.	84	M	Y/L	HT, glaucoma	Tumor mass on right side of mouth	SMG (R)	Painless, soft, movable tumor, ∼ 2 × 2 cm in diameter	Metastatic	N	11 y	N	CCC
243.	60	M	Y/R	DM, HT	Gum mass	G (max, L, ant)	Exophytic, erythematous lesion with a granulomatous appearance, 2 cm in size	PG, PGCG, SCC, Metastatic carcinoma	N	5 y	N	CCC
244.	59	M	Y/ R	N	Rapidly growing painless nodule for 1 mo	LL	Nodule crossing vermillion border	PG, SCC, Metastatic carcinoma	N	7 y	N	CCC
245.	75	F	Y/R	DM, cardiac arrhythmia, pacemaker	Bleeding gums for 10 d and tumor-like growth	G (max L, ant)	Facial asymmetry, deformed left UL, tumor mass in 22–23 region, 22 missing, mobile 21, 23	PGCG	N	2 y	Multiple	CCC
246.	54	M	N	HT, nicotine	Painless rapidly growing mass on left preauricular region for 6 mo	P (L)	Palpable mass with lymphadenopathy	SGT	Y	–	Lung, neck, rib	CCC
247.	58	M	Y/R	DM, HT, hypothyroidism	Loss of appetite, disorientation, drowsiness, and ulcer in the mouth	BM (L)	Ulcerated growth	NA	N	1 y	Brain, scalp	CCC
248.	75	F	N	HT, DM, colon cancer, CAD, diabetic nephropathy	Painful swelling on left preauricular region	P (L)	Firm, nodular growth	SGT	Y	–	Vertebrae	CCC
249.	59	M	Y/R	N	Preauricular swelling right side	P (R)	6 × 4 cm nodular, vascular, mass	NA	N	4 y	N	CCC
250.	52	M	Y/NA	S, HT, DM, IHD	Sore throat for 2 wk	Uvula	Protruding mass	Polyp, benign, or malignant oral tumor	N	1 y	Lung, liver, skeletal	CCC

Abbreviations: A, alcohol; Ant, anterior; AN, areca nut; AS, angiosarcoma; BA, bronchial asthma; BL, bilateral; BM, buccal mucosa; BOP, bleeding on probing; BPA, benign prostate atrophy; COPD, chronic obstructive pulmonary disease; CRF, chronic renal failure; DM, diabetes mellitus; F, female; FNP, facial nerve palsy; G, gingiva; GBS, Guillain Barr syndrome; HOE, history of extraction; HP, hard palate; HT, hypertension; I, ischemic heart disease; L, left; LC, lung cancer; LL, lower lip; M, male; MS, multiple sites; N, no; NA, not available; OSTT, oral soft tissue tumor; P, parotid; HP, hard palate; KA, keratoacanthoma; KS, Kaposi sarcoma; PG, pyogenic granuloma; PGCG, peripheral giant cell granuloma; Post, posterior; R, right; RCC, renal cell carcinoma; S, smoking; SCC, squamous cell carcinoma; SGT, salivary gland tumor; SM, skeletal muscles; SMG, submandibular gland; SNA, site not available; SP, soft palate; T, tongue; TCC, transitional cell carcinoma; To, tonsil; TN, trigeminal neuralgia; UL, upper lip; UTI, urinary tract infection; Y, yes; y, years.

**Table 3:** Data describing treatment and prognosis of patients with renal cell carcinoma metastasizing to the oral soft tissues (1911–2022)

Patient no.	Treatment done	Prognosis	Survival time from DOM to D (in months)	Reason for death
1.	NA	NA	NA	NA
2.	NG	D	3	NA
3.	Died before diagnosis, diagnosed at autopsy	–	–	–
4.	NG	D	1	MM
5.	NA	NA	NA	NA
6.	NA	NA	NA	NA
7.	S	D	5	NA
8.	NA	NA	NA	NA
9.	NA	NA	NA	NA
10.	NA	NA	NA	NA
11.	S, R	NA	NA	NA
12.	NA	NA	NA	NA
13.	S	NA	NA	NA
14.	NA	NA	NA	NA
15.	NG	D	1	NA
16.	NA	Fav/Alive	–	–
17.	NA	D	1	NA
18.	E, R	FAV	–	–
19.	S	NA	NA	NA
20.	S	D	3	NA
21.	Systematic	Fav	–	–
22.	NA	NA	NA	NA
23.	NA	NA	NA	NA
24.	NA	NA	NA	NA
25.	Superficial parotidectomy	NA	NA	NA
26.	NA	NA	NA	NA
27.	S, R	D	3	NA
28.	NA	NA	NA	NA
29.	S	Fav	–	–
30.	NA	NA	NA	NA
31.	Deep parotidectomy	NA	NA	NA
32.	NA	NA	NA	NA
33.	S	NA	NA	NA
34.	Cryotherapy	NA	NA	NA
35.	S	NA	NA	NA
36.	NA	NA	NA	NA
37.	Superficial parotidectomy	NA	NA	NA
38.	S	Fav	–	–
39.	S	NA	NA	NA
40.	R, C	D	7	NA
41.	S	NA	NA	NA
42.	C, I	D	2	NA
43.	S	D	NA	NA
44.	NA	NA	NA	NA
45.	NA	NA	NA	NA
46.	Partial parotidectomy	NA	NA	NA
47.	NA	NA	NA	NA
48.	Palliative radiotherapy	NA	NA	NA
49.	NA	NA	NA	NA
50.	Superficial parotidectomy	NA	NA	NA
51.	Complete parotidectomy	NA	NA	NA
52.	Superficial parotidectomy	D	20	NA
53.	S, R	D	46	NA
54.	NA	NA	NA	NA
55.	NA	NA	NA	NA
56.	NA	NA	NA	NA
57.	Superficial parotidectomy	NA	NA	NA
58.	Parotidectomy	NA	NA	NA
59.	S	D	6	Hepatic insufficiency
60.	NA	NA	NA	NA
61.	Superficial parotidectomy	NA	NA	NA
62.	S	D	3	NA
63.	Superficial parotidectomy	Fav	–	–
64.	I	D	6	MM
65.	NA	NA	NA	NA
66.	S, R, I	D	6	NA
67.	S	D	2	NA
68.	Partial parotidectomy	NA	NA	NA
69.	NA	NA	NA	NA
70.	NA	NA	NA	NA
71.	Superficial parotidectomy	NA	NA	NA
72.	S	D	36	MM
73.	NA	NA	NA	NA
74.	S	NA	NA	NA
75.	S, I	Fav	–	–
76.	Superficial parotidectomy	NA	NA	NA
77.	NA	NA	NA	NA
78.	R	D	12	RF
79.	Complete parotidectomy	Fav	–	–
80.	S	NA	NA	NA
81.	R, C, I, IL	D	16	Lung metastasis
82.	Tumor resection	Fav	–	–
83.	R	NA	NA	NA
84.	NA	NA	NA	NA
85.	NA	NA	NA	NA
86.	Parotidectomy (TNA) and nephrectomy	NA	NA	NA
87.	NA	NA	NA	NA
88.	Superficial parotidectomy	NA	NA	NA
89.	E	NA	NA	NA
90.	C	NA	NA	NA
91.	S, I, IL	NA	NA	NA
92.	S, R	D	NA	NA
93.	Superficial parotidectomy	NA	NA	NA
94.	Palliative, blood transfusion	D	NA	Uncontrolled bleeding
95.	S, I	Fav	–	–
96.	S	D	5	MM
97.	NA	NA	NA	NA
98.	Superficial parotidectomy	NA	NA	NA
99.	R	D	NA	RF
100.	S, C	NA	NA	NA
101.	S	D	1	MM
102.	Superficial parotidectomy	NA	NA	NA
103.	Parotidectomy	NA	NA	NA
104.	S, R, C, I	Fav	–	–
105.	S	D	6	NA
106.	S	NA	NA	NA
107.	NA	NA	NA	NA
108.	NA	NA	NA	NA
109.	S	Fav	NA	NA
110.	S	NA	NA	NA
111.	S	NA	NA	NA
112.	Superficial parotidectomy	NA	NA	NA
113.	Tumor resection	D	12	DC
114.	S, R, I	Fav	–	–
115.	R, I	D	2	NA
116.	NG	–	–	–
117.	Superficial parotidectomy	NA	NA	NA
118.	NA	NA	NA	NA
119.	Superficial parotidectomy	NA	NA	NA
120.	R, palliative	NA	NA	NA
121.	Complete parotidectomy, R	NA	NA	NA
122.	NG	D	NA	MM
123.	S	NA	NA	NA
124.	Complete parotidectomy, further Tt RBP	Fav	–	–
125.	E	NA	NA	NA
126.	E	Fav	NA	NA
127.	Superficial parotidectomy	NA	NA	NA
128.	S, R	Fav	–	–
129.	NA	NA	NA	NA
130.	NA	NA	NA	NA
131.	S, R	D	5	NA
132.	NA	NA	NA	NA
133.	E, I	NA	NA	NA
134.	S	NA	NA	NA
135.	S, R	D	12	NA
136.	S, C	D	9	MM
137.	S, palliative	NA	NA	NA
138.	Superficial parotidectomy	Fav	NA	NA
139.	R	D	12	NA
140.	Palliative	D	3	NA
141.	Superficial parotidectomy	NA	NA	NA
142.	S	Fav	–	–
143.	Palliative	D	2	NA
144.	Palliative radiotherapy	Fav	–	–
145.	S	D	24	NA
146.	S	D	3	NA
147.	Complete parotidectomy, R, C	Fav	–	–
148.	Embolization	D	NA	RF
149.	S	D	5	NA
150.	S, R	Fav	–	–
151.	Superficial parotidectomy	NA	NA	NA
152.	Complete parotidectomy	Fav	–	–
153.	Cryosurgery	D	12	NA
154.	S, R, I	NA	NA	NA
155.	Superficial parotidectomy	Fav	–	–
156.	S, R	Fav	–	–
157.	Brachytherapy	D	5	MM
158.	I	NA	NA	NA
159.	R	NA	NA	NA
160.	S	Fav	–	–
161.	IL	UFU	–	–
162.	S	Fav	–	–
163.	Complete parotidectomy, further Tt refused by patient	NA	NA	NA
164.	NA	NA	NA	NA
165.	S	NA	NA	NA
166.	S, C	UFU	–	–
167.	C, R, conservative	UFU	–	–
168.	Parotidectomy with preservation of FN, R	UFU	–	–
169.	Parotidectomy (partial), nephrectomy, sunitinib	Fav	–	–
170.	S, C	TGO	–	–
171.	S, R	Fav	–	–
172.	Complete parotidectomy, adrenalectomy, nephrectomy	Fav	–	–
173.	Systematic	NA (size increased)	–	–
174.	I	NA	NA	NA
175.	Total parotidectomy sacrificing FN, RND, and hemithyroidectomy with isthmectomy	NA	–	–
176.	S, C, R	UFU	–	–
177.	Superficial parotidectomy	Fav	–	–
178.	S	NA	NA	NA
179.	NA	NA	NA	NA
180.	Superficial parotidectomy	Fav	–	–
181.	R, TKI	Fav	–	–
182.	NA	NA	NA	NA
183.	S, I	NA	–	–
184.	NA	NA	NA	NA
185.	R, TKI, S	Fav	–	–
186.	Palliative radiotherapy	NA	NA	NA
187.	Complete parotidectomy	NA	NA	NA
188.	Complete parotidectomy	NA	NA	NA
189.	NA	NA	NA	NA
190.	Tumor resection	NA	NA	NA
191.	Superficial parotidectomy	NA	NA	NA
192.	Complete parotidectomy	NA	NA	NA
193.	Tumor resection	NA	NA	NA
194.	Complete parotidectomy	NA	NA	NA
195.	Tumor resection	NA	NA	NA
196.	R, S	Fav	–	–
197.	Complete parotidectomy	NA	NA	NA
198.	NA	NA	NA	NA
199.	NA	D	6	NA
200.	Sunitinib (targetoid)	TGO	–	–
201.	C	NA	NA	NA
202.	R, TKI	D	1	MM
203.	S, R	Fav	–	–
204.	S	D	3	NA
205.	Planned for S, C, (NG)	NA	NA	NA
206.	Systemic	D	10 d	Hemorrhage
207.	NA	NA	NA	NA
208.	S, R, C	D	6	MM
209.	Parotidectomy with preservation of FN	NA	NA	NA
210.	Parotidectomy	Fav	–	–
211.	Symptomatic therapy	NA	NA	NA
212.	S, R	Fav	–	–
213.	S, R, C, TKI	NA	NA	NA
214.	C, conservative	Fav	–	–
215.	S	Fav	–	–
216.	S, R	D	8	MM
217.	RTO	NA	NA	NA
218.	Systematic	Fav	–	–
219.	Palliative R	D	21 d	NA
220.	Tonsillectomy	NA	NA	NA
221.	C, R	Fav	–	–
222.	E, palliative radiotherapy	TGO	–	–
223.	C, R	D	4	NA
224.	Partial parotidectomy	UFU	–	–
225.	Planned for R, C	D	12	NA
226.	Palliative	D	20 d	NA
227.	RBP	D	12	NA
228.	S, C, R	D	6	NA
229.	S	Fav	–	–
230.	S	Fav	–	–
231.	E, RTO	D	NA	NA
232.	RTO	UFU	–	–
233.	Systematic	Fav	–	–
234.	RTO	UFU	–	–
235.	CT, C	D	1	NA
236.	E	Fav	–	–
237.	Targetoid therapy	NA	NA	NA
238.	NA	NA	NA	NA
239.	Parotidectomy	Fav	–	–
240.	Parotidectomy	NA	NA	NA
241.	Radical excision of gland	Fav/TGO	–	–
242.	Radical excision of gland	Fav/TGO	–	–
243.	S	Fav	–	–
244.	Lost to follow-up	–	–	–
245.	E	D	15	NA
246.	Nephrectomy	TGO	–	–
247.	R, systematic	UFU	–	–
248.	T, R	TGO	–	–
249.	Targetoid	UFU	Fav/Alive	–
250.	E, R, palliative immunotherapy	Fav, UFU		–

Abbreviations: C, chemotherapy; CT, cryotherapy; D, death; d, days; DC, deteriorated condition; DOM, diagnosis of metastasis; E, excision; Fav, favorable; I, interferon; IL, interleukin; MM, multiple metastasis; NA, not available; NG, not given; RBP, refused by patient; RF, respiratory failure; RTO, referred to oncologist; S, surgery; TGO, treatment going on; Tt, treatment; UFU, under follow-up.

## Results

Our research strategy revealed a total of 226 relevant articles. 11 12 13 14 15 16 17 18 19 20 21 22 23 24 25 26 27 28 29 30 31 32 33 The results of the current research were expressed in descriptive statistics. A total of 250 patients were included with 168 males and 67 females with a male to female ratio of 2.5:1. The maximum number of cases were from the United States ( *n* = 54) followed by Japan ( *n* = 28), United Kingdom ( *n* = 22), Turkey ( *n* = 18), Italy ( *n* = 17), India = Poland ( *n* = 16), and Spain ( *n* = 14). The patients' average age was 62.7 years (range: 18–97). Mean age was 62.4 years in males and 63.7 years in Females. Of the 250 patients, 126 (50.4%) had a previous history of RCC, while 79 had none (31.6%). The most predominant site of OSTM was salivary glands (39.2%) followed by tongue (27.2%) and gingiva (16%). OST was the initial site of metastasis in 31.2% of individuals, the only site of metastasis in 57.2% of cases, whereas 24.8% of cases involved other distant sites too. The most common type of RCC diagnosed was clear cell carcinoma (CCC). Major therapeutic aids included were surgery (41.2%) and combined therapies (22%) ( Table 4 ). Twenty-three percent of patients died with a mean survival rate of 10 days to 4 years.

**Table 4:** Summary of results documented from literature research describing the characteristics of renal cell carcinoma metastasizing to the oral soft tissues (1911–2022)

Feature	Number
Total number of articles published	226 • Case reports—198 • Case series—9 • Retrospective analysis—8 • Original research—3 • Clinicopathological study—3 • Systematic reviews—2 • Correspondences—2 • Prospective study—1
Total number of patients	250
World-wide distribution of cases	• USA—54 (21.6%) • Japan—28 (11.2%) • UK—22 (8.8%) • Turkey—18 (7.2%) • Italy—17 (6.8%) • India = Poland—16 (6.4%) • Spain—14 (5.6%) • Germany—9 (3.6%) • Europe—7 (2.8%) • Brazil—6 (2.4%) • France—5 (2%) • Australia = Finland = Greece = Iran—4 (1.6%) • China—3 (1.2%) • Israel = Saudi Arabia—2 (0.8%) • Africa = Argentina = Chile = Czech Republic = Jordan = Morocco = the Netherland = Ireland = Romania = Russia = Sri Lanka = Sudan = South Korea = Canada = Taiwan—1 (0.4%)
Gender	• M—168 (67.2%) • F—67 (26.8%) • NA—15 (6%) M:F = 2.5:1
Average age of patients (range)	• Total—62.7 y (18–97 y) • Males—62.4 y (27–86 y) • Females—63.7 y (18–97 y)
Previous history of RCC	• Yes—126 (50.4%) R—35 (27.7%) L—48 (38.1%) NA—43 (34.1%) • No—79 (31.6%) • NA—45 (18%)
Associated risk factors	• Yes—114 (45.6%) • No—85 (34%) • NA—51 (20.4%) • Hypertension—14 (12.3%) • Smoking—12 (10.5%) • Renal = other malignancies—7 (6.1%) • Alcohol = cardiac = DM = respiratory—4 (3.5%) • MM—3 (2.6%) • Tobacco = anemia = thyroid = prostate—2 (1.7%) • Areca nut = HOE = FNP = family history of RCC = recurrent RCC—1 (0.8%) • Others—10 (8.8%)
Site of oral metastasis	• Salivary glands—98 (39.2%) o Parotid—84 (85.7%) (R—36, L—32, BL—3, SNA—13) o Submandibular—12 (12.2%) (L—4, R—6, BL—0, SNA—2) o Minor glands retromolar—1 (1%) o Wharton duct—1 (1%) • Tongue—68 (27.2%) (base—12, dorsum—9, tip—6, ant—3, post—2, L—6, R—6, body—1, SNA—23) • Gingiva—40 (16%) (maxillary—15, mandibular—12, SNA—13) o Maxillary—(ant—6, post—1, SNA—8) (R—2, L—3, both—3, SNA—7) o Mandibular (ant—3, post—4, both—2, SNA—3) (R—3, L—2, both—2, SNA—5) • Lip—12 (4.8%) (upper—5, lower—6, SNA—1) • Buccal mucosa—11 (4.4%) (R—2, L—7, NA—2) • Hard palate—8 (3.2%) • Palatine tonsils—6 (2.4%) • Uvula—3 (1.2%) • Chin—3 (1.2%) • Soft palate—2 (0.8%) • Retromolar region—1 (0.4%) • Facial skin—1 (0.4%) • Cheek—1 (0.4%) • SNA—1 (0.4%)
Oral soft tissues as the initial site of metastasis	• Yes—78 (31.2%) • No—128 (51.2%) • Detected at the same time—1 (0.4%) • NA—43 (17.2%)
Any other site of metastasis	• Yes—62 (24.8%) • No—143 (57.2%) • NA—45 (18%)
Average time of detection of metastasis after history of nephrectomy	3 weeks to 26 years
Type of RCC	• CCC—241 (96.4%) • Papillary carcinoma—1 (0.4%) • Collecting duct carcinoma—1 (0.4%) • TCC of renal pelvis—1 (0.4%) • NA—6 (2.4%)
Treatment aids	• Surgical aids—103 (41.2%) (parotidectomy—48, not specified—43, excision—8, tumor resection—6) • Combined therapy—55 (22%) • Radiotherapy—5 (2%) • Systematic—5 (2%) • Interferon—3 (1.2%) • Palliative radiotherapy—3 (1.2%) • Palliative treatment—3 (1.2%) • Symptomatic—2 (0.8%) • Targetoid therapy—2 (0.8%) • Chemotherapy—1 (0.4%) • Cryotherapy—1 (0.4%) • Cryosurgery—1 (0.4%) • Brachytherapy—1 (0.4%) • Interleukins—1 (0.4%) • Embolization—1 (0.4%) • Refused by patient—1 (0.4%) • Referred to oncologist—4 (1.6%) • Tt planned but died before—1 (0.4%) • Tt planned but further information NA—1 (0.4%) • NA—50 (20%) • NG—6 (2.4%)
Prognosis	• Deaths—57 (23%) • Favorable—48 (19%) • UFU—11 (4.4%) • TGO—7 (2.8%) • LFU—1 (0.4%) • NA—125 (50%)
Average time of death from diagnosis of oral metastasis	10 days to 4 years

Abbreviations: Ant, anterior; BL, bilateral; DM, diabetic mellitus; F, female; HOE, history of extraction; L, left; LFU, lost to follow-up; M, male; MM, multiple metastasis; NA, not available; NG, not given; post, posterior; R, right; RBP, refused by patient; RCC, renal cell carcinoma; SNA, site not available; TGO, treatment going on; Tt, treatment; UFU, under follow-up; Y, yes; y, years.

## Discussion

Metastasis to the oral cavity is a rare occurrence, with the real incidence unclear (1–2% of all oral cancers). Because of their rarity, they are often overlooked for a long time before being discovered and are diagnosed during investigations. RCC is the third most common tumor metastasizing to oral cavity after lung, and liver cancer. 34 In the current research, we found 250 cases of primary RCC metastasizing to OST.

RCC is the most common solid renal tumor originating from the proximal renal tubular epithelium. Worldwide, 403,000 new cases of RCC and 175,000 deaths due to this malignancy were recorded in 2018. In India, the incidence of RCC among males is about 2/100,000 population and among females is about 1/100,000 population. 35 RCC has rapidly become more common in the developed world over the past decades, 36 more than doubling in incidence in the United States since 1975. In our research also, the maximum number of cases were from the United States followed by Japan, United Kingdom, Turkey, India, Spain, Poland, and Europe ( Table 4 ).

RCC occurs predominantly during fifth to sixth decades exhibiting a male predilection with a male:female ratio of 1.5:1. In the current study also, there was a male predominance, with a male:female ratio = 2.5:1. However, the age ranged between first and ninth decades.

Multiple risk factors favor the development of RCC which include smoking, tobacco chewing, alcohol, obesity, hypertension, cardiac, liver and renal diseases, urinary stones, diabetes, drug usage, and malnutrition. 2 Studies have reported that cigarette smoke contains many carcinogens as well as the highly addictive substance called nicotine. As they are filtered through the nephron, these particles are metabolized and promote inflammation and induce DNA damage, paving the way for carcinogenesis. Smokers are known to exhibit more risk of RCC than non-smokers. 37 However, in the current research, only 10.5% of cases have revealed the habit of smoking. Many had variable comorbidities, including renal, cardiac, and endocrinal diseases, and history of other malignancies. One patient revealed the family history of RCC.

Pathogenic mechanisms of metastasis to the OST are not completely recognized. Route of secondary metastasis may be hematogenous, lymphatic, or direct invasion. Metastatic RCC spreads predominantly following the hematogenous route. One of the proposed pathways is via Batson's valve plexus system. Angiogenesis plays a crucial role in the development of tumor metastasis. The tumor-derived micro vesicles break off from the primary site. These micro vesicles appear to carry a cancer stem cell phenotype and microRNAs which stimulate angiogenesis. 4 RCC is naturally a pre-angiogenetic cancer. It is hypothesized that the kidneys receive about 25% of the circulating blood volume per minute, in addition to the release of vascular endothelial growth factor and other angiogenic factors by RCC, resulting in the hypervascularity of these tumors and their association with arteriovenous shunts contributing to the unique hematogenous route of spread. Majority of cases of RCC involve dysfunction of Von–Hippel–Lindau gene which promotes ubiquitination and inactivation of hypoxia-induced factor in healthy individuals which creates a pre-angiogenic environment. 4

The OST has a rich capillary network, and the uneven basement membrane of proliferating capillaries may allow malignant cells to penetrate the tissues more easily.

The most common site of OSTM is the attached gingiva (57%), followed by the tongue (27%), tonsil (8%), palate (4%), lip (3%), buccal mucosa (BM) (1%), and floor of mouth (<1%). 9 OSTM of the RCC mostly affects tongue, gingiva, and parotid glands. 5 In the current study, the majority of RCC metastasis to OST were found in salivary glands (39.2%), tongue (27.2%), gingiva (16%), lip (4.8%), BM (4.4%), HP (3.2%), palatine tonsils (2.4%), uvula (1.2%), soft palate (SP) (0.8%), and in the retromolar region = minor glands = Wharton duct = facial skin = cheek (0.4%). And the results were compared with the previous reviews ( Table 5 ). Malignant neoplasms of the salivary glands are very rare with 1 to 4% occurrence and the parotid gland being the most affected. Approximately 0.1% of all salivary gland metastatic neoplasms exhibit a primary focus to be RCC. In parotid, RCC metastasis is mainly through hematogenous route due to high vascularity of these lesions. Udager and Rungta and Lieder et al published a review of the literature reporting 36 and 45 cases of RCC parotid metastasis. 12 13 In our research, 98 of 250 cases involved the salivary glands, with maximum instances involving the parotid (84/240). SMG involved only 12 cases. None of the cases involved sublingual gland. One case affected solely the Wharton duct. While in another case, only minor salivary glands of retromolar region were involved. The tongue is a highly circulatory organ, which creates ideal conditions for the spread of cancer. Posterolateral and dorsal part are more often involved in metastasis due to rich capillary and lymphatic network and immobility. Irani in 2016 documented that in 19 of 58 cases of RCC, metastatic site was the tongue. 5 In the current research, 67 of 250 cases of metastatic RCC involved the tongue, maximally affecting the base with 12 cases followed by dorsum, tip, and lateral border. Chronically, inflamed mucosa of gingiva, particularly the attached gingiva, contains a dense capillary network that can trap malignant cells and promote metastases. In the current research, gingiva was the third most common site of RCC metastasis (16% cases). Studies conclude that gingival metastasis mostly occurs in mandibular area than in maxillary with predominancy of posterior side involvement. 5 6 7 8 In the current research, however, there was maxillary predilection. Anterior region was mostly affected in maxilla, whereas there was almost equal involvement of anterior and posterior sides in mandible. The extraction site of tooth is thought to be a microenvironment rich in local growth factors that encourage metastatic cell development. 5 In our research, one peculiar case of post-extraction site metastasis has been observed in which patient approached with a complaint of painful growth in the region of extracted tooth after 7 days. Lip, BM, HP, SP, uvula, and tonsils are the rare sites of OSTM from distant resources. Few cases of lip metastasis have been reported from colon and gastric cancers. In the present review, only 12 cases of lip and 11 cases of BM from RCC were notified. The most common malignant neoplasms of the palatal mucosa are known to be minor salivary gland tumors and metastatic tumor from a distant organ in this region is uncommon. 38 In the present research, only 13 cases were found in the palate region. According to a research, only 0.8% of malignant palatine tonsillar tumours was from an extra-tonsillar source. 39 Lymphatic spread to tonsils is rare due to lack of afferent lymphatic capillaries except retrograde spread via cervical lymph nodes or direct spread; thus, metastatic pathway is unclear. In the current literature, only six cases of palatine tonsillar metastasis from RCC have been observed.

**Table 5:** Comparison of sites of oral soft tissues metastasis from renal cell carcinoma with the previous reviews conducted in the literature

Author, year	Sujonin et al, 2014	Vasilyeva et al, 2017	Kovalski et al, 2020	Nisi et al, 2020	Current study, 2022
**Duration**	1975–2014	2007–2017	(2010–2019)	(1911–2020)	(1911–2022)
**Oral sites affected (number)**	• P—10 • T—8 • OM—6 • To, facial muscles, oropharynx—9	• T—12 • G—8 • L—2 • HP—1 • SP—1 • BM—1	• T—11 • G—8 • SMG—2 • P—8 • L—2 • BM—1 • Cheek—2	• T—56 • G—26 • L—6 • HP—4 • SP—1 • BM—1	• P—84 • T—68 • G—40 • L—12 • BM—11 • SMG—12 • HP—8 • To—6 • Uvula—3 • SP—2 • Chin—2 • Retromolar—1 • Minor glands—1 • Wharton duct—1 • Facial skin—1
**Reference no.**	11	14	18	19	–

Abbreviations: BM, buccal mucosa; G, gingiva; HP, hard palate; L, lip; P, parotid; SMG, submandibular gland; SP, soft palate; T, tongue; To, tonsils.

Oral metastatic tumours are of high clinical importance because they may be the only symptom of an undiagnosed underlying malignancy or the first sign of the metastasis. In our study, 31.2% cases of OSTM from RCC presented as the initial site of metastasis, whereas in 51.2% cases, metastasis was detected after the nephrectomy done for RCC, with an average time of 3 weeks to 26 years. The clinical aspects of kidney metastasis in the OST vary according to the anatomical site involved characterized by rapidly growing painful or asymptomatic swellings that bleed easily, difficulty in chewing, and dysphagia. One of the characteristic features of metastatic RCC is their intense vascularization. These metastatic lesions often become difficult to diagnose because their variable appearances bear close resemblance to some benign hyperplastic or reactive oral lesions. In the present research, rapidly increasing vascular swelling was the most predominant clinical feature observed. Other lesions appeared as ulcerative, exophytic, pedunculated, nodular, and edematous. A history of primary tumor could help in the detection of secondary metastatic deposits. Before the metastatic spread to the oral cavity, the majority of patients are aware of their primary tumours. However, metastasis to OST via RCC is a late indication. In the current research, 50.4% of patients had a previous history of primary RCC with nephrectomy; 31.6% of patients did not reveal such history.

Histopathological examination is required to provide a conclusive diagnosis of the type of metastatic lesion. However, it might be difficult to make an exact diagnosis because of varied histological appearance, particularly when the major focus of primary site is unknown. Other tools, such as special staining, immunohistochemistry, and electron microscopy, may be necessary in some circumstances to determine the initial tumor's nature. Histopathologically, RCC has been divided into various subgroups. The World Health Organization classification of urogenital tumors in 2022 have introduced many new entities in the RCC. 40 CCC is the most predominant type and has been discovered to be the most prevalent metastasizing to the OST. The finding was same in this study as well. 96.4% cases were diagnosed to be CCC. Although RCC entails multiorgan distant metastases, OST might occasionally be the only site of metastasis. Out of 250 instances in this study, 143 had OST as the only location of RCC metastasis, whereas 62 had metastasis to other regions as well, such as lungs, brain, adrenals, liver, vertebrae, spine, pelvis, skin, and skeletal muscles.

Treatment options for metastatic RCC include biopsy, surgery, chemotherapy, radiotherapy, brachytherapy, and/or combination therapy. The most commonly used therapeutic aids in this study were surgical aids (41.2%) and combination therapy (22%). Salivary gland lesions were treated by parotidectomy, superficial, deep, partial, or total depending on the site. Unfortunately, OSTM by RCC has a bad prognosis with a maximum survival rate of approximately 5 years. In some cases, a patient's terminal stage of disease results in a loss of follow-up or death. Even after treatment, 57 people died, according to the current study with a survival time of 10 days to 4 years. Multiple metastases, deteriorated systemic condition, hepatic insufficiency, and uncontrolled bleeding were the most common causes of death. Forty-eight patients had a good prognosis with no signs of recurrence. In seven patients, treatment is going on. Eleven cases are under follow-up.

## Limitations of the Current Study

One of the limitations of current research was small sample size. Most of the included studies were case reports and case series. And in many of the included studies, individual data of patients were not available.

## Conclusions

During the last 111 years (1911–2022), we found only 250 cases of OSTM from RCC as the sole primary source. This signifies a rare occurrence of OSTM from RCC. The prognosis was poor involving 23% deaths with a survival rate of few days to 4 years. Parotid, tongue, and gingiva were the most prevalent sites to get metastasized. Because of their resemblance to other pathologies, and late clinical signs, these lesions go unnoticed the majority of the time. Diagnosis of oral metastatic lesions is a challenging task for the clinicians and pathologists. A thorough examination of the metastatic lesions is required, including a review of the patient's medical history, clinical presentation, and early diagnosis in order to identify the primary site of metastasis and choose the best course of treatment.

## References

[1] Bray F Ferlay J Soerjomataram I Siegel R L Torre L A Jemal A Global cancer statistics 2018: GLOBOCAN estimates of incidence and mortality worldwide for 36 cancers in 185 countries CA Cancer J Clin 2018 68 06 394 424 30207593 10.3322/caac.21492

[2] Padala S A Barsouk A Thandra K C Epidemiology of renal cell carcinoma World J Oncol 2020 11 03 79 87 32494314 10.14740/wjon1279 PMC7239575

[3] Brufau B P Cerqueda C S Villalba L B Izquierdo R S González B M Molina C N Metastatic renal cell carcinoma: radiologic findings and assessment of response to targeted antiangiogenic therapy by using multidetector CT Radiographics 2013 33 06 1691 1716 24108558 10.1148/rg.336125110

[4] Gong J Maia M C Dizman N Govindarajan A Pal S K Metastasis in renal cell carcinoma: biology and implications for therapy Asian J Urol 2016 3 04 286 292 29264197 10.1016/j.ajur.2016.08.006 PMC5730828

[5] Irani S Metastasis to the oral soft tissues: a review of 412 cases J Int Soc Prev Community Dent 2016 6 05 393 401 27891304 10.4103/2231-0762.192935 PMC5109852

[6] Hirshberg A Shnaiderman-Shapiro A Kaplan I Berger R Metastatic tumours to the oral cavity - pathogenesis and analysis of 673 cases Oral Oncol 2008 44 08 743 752 18061527 10.1016/j.oraloncology.2007.09.012

[7] Servato J P de Paulo L F de Faria P R Cardoso S V Loyola A M Metastatic tumours to the head and neck: retrospective analysis from a Brazilian tertiary referral centre Int J Oral Maxillofac Implants 2013 42 11 1391 1396 10.1016/j.ijom.2013.05.020 23870760

[8] Hirshberg A Berger R Allon I Kaplan I Metastatic tumors to the jaws and mouth Head Neck Pathol 2014 8 04 463 474 25409855 10.1007/s12105-014-0591-z PMC4245411

[9] Riley D S Barber M S Kienle G S CARE guidelines for case reports: explanation and elaboration document J Clin Epidemiol 2017 89 218 235 28529185 10.1016/j.jclinepi.2017.04.026

[10] STROBE Initiative von Elm E Altman D G Egger M Pocock S J Gøtzsche P C Vandenbroucke J P The strengthening the reporting of observational studies in epidemiology (STROBE) statement: guidelines for reporting observational studies J Clin Epidemiol 2008 61 04 344 349 18313558 10.1016/j.jclinepi.2007.11.008

[11] Suojanen J Färkkilä E Helkamaa T Rapidly growing and ulcerating metastatic renal cell carcinoma of the lower lip: a case report and review of the literature Oncol Lett 2014 8 05 2175 2178 25289097 10.3892/ol.2014.2505 PMC4186528

[12] Udager A M Rungta S A Metastatic renal cell carcinoma, clear cell type, of the parotid gland: a case report, review of literature, and proposed algorithmic approach to salivary gland clear cell neoplasms in fine-needle aspiration biopsies Diagn Cytopathol 2014 42 11 974 983 24535952 10.1002/dc.23103

[13] Lieder A Guenzel T Lebentrau S Schneider C Franzen A Diagnostic relevance of metastatic renal cell carcinoma in the head and neck: an evaluation of 22 cases in 671 patients Int Braz J Urol 2017 43 02 202 208 27649110 10.1590/S1677-5538.IBJU.2015.0665 PMC5433357

[14] Vasilyeva D Peters S M Philipone E M Yoon A J Renal cell carcinoma metastatic to the maxillary gingiva: a case report and review of the literature J Oral Maxillofac Pathol 2018 22 01 S102 S107 29491617 10.4103/jomfp.JOMFP_69_17 PMC5824500

[15] Sydney G Ioakim K Kara N George Pantelas G Rare case of clear cell renal cell carcinoma metastasizing to contralateral kidney and ipsilateral parotid more than five years following nephrectomy Balk J Dent Med 2019 23 108 111

[16] Fejsa Levakov A Amidžic J Ilić Sabo J Lakić T Vojinov S Grbić D Unusual site for metastatic renal cell carcinoma – a case report Vojnosanit Pregl 2020 7 233 236

[17] Halbony H Albrezat M Hmaid D Albsoul N Parotid gland metastasis as an initial presentation of renal cell carcinoma: a case report Med J Islam Repub Iran 2020 34 175 33816374 10.47176/mjiri.34.175 PMC8004575

[18] Kovalski L NS Ribeiro J T Martins M D A rare case of oral metastasis of renal clear cell carcinoma: case report and review of literature J. Oral Diag. 2020 05 e20200006

[19] Nisi M Izzetti R Graziani F Gabriele M Renal cell carcinoma metastases to the oral cavity: report of 2 cases and review of literature J Oral Maxillofac Surg 2020 78 09 1557 1571 32386976 10.1016/j.joms.2020.04.001

[20] Patel S Barros J Nwizu N N Ogbureke K UE Metastatic renal cell carcinoma to the oral cavity as first sign of disease: a case report Clin Case Rep 2020 8 08 1517 1521 32884786 10.1002/ccr3.2923 PMC7455456

[21] Stojanović M Krasić D Trajković M Petrović V Rare renal cell carcinoma metastasis to mandibular gingiva: a case report and literature review Niger J Clin Pract 2020 23 10 1483 1486 33047710 10.4103/njcp.njcp_55_19

[22] Cecen A Kavaz E Gun S A rare case: renal cell carcinoma metastasis to lower lip J Exp Clin Med 2021 38 396 397

[23] Chelliah P Shah K M Vandergriff T Nijhawan R I Pink nodule of the chin: an unusual presentation of metastatic carcinoma Dermatol Online J 2021 27 08 2 5 10.5070/D327854713 34755966

[24] Darshan D P Rahul A Umank B T Unusual site of metastasis in a case of renal cell carcinoma - a case report Guj Canc Soc Res J 2021 1 25 27

[25] Gopan G Kamala L H Radhakrishnan N Renal cell carcinoma presenting as bulky parotid mass - a case report and review of literature Indian J Surg Oncol 2021 12 02 378 382 10.1007/s13193-021-01413-6 PMC8716801 35035177

[26] Martire M B Villena L F Sousa J A Jr Montoro J RM Uvo S AB Parotid metastasis of clear-cell renal cell carcinoma (ccRCC): a case report Arch Head Neck Surg. 2021 50 e20215016

[27] Santana T Custódio M Dayla Melo Oliveira C Dos Santos Antunes E Cantanhede Orsini Machado de Sousa S Daumas Nunes F Parotid metastasis of clear cell renal cell carcinoma 8 years after nephrectomy Oral Oncol 2021 122 105561 34634669 10.1016/j.oraloncology.2021.105561

[28] Villanueva F Fonseca D Rojas C Escalante l. Granulomatous lesion in inserted gum-metastasis of renal clear ODOVTOS-Int J Dental Sc 2021 23–1 43 52

[29] Williams J Depcik-Smith N Williams T Feldman S R Metastatic renal clear cell carcinoma masquerading as a pyogenic granuloma on the lip Dermatol Online J 2021 27 11 1 6 10.5070/D3271156090 35130402

[30] Baez F S Collazo P Oral metastasis of renal cell carcinoma. A case report and literature review Odontoestomatologia 2022 24 1 7

[31] Lavanya M L Iyer P JM Vijayakumar R Buccal mucosal metastasis of renal cell carcinoma: a case report and review of literature International Journal of Clinical Research 2022 3 132 137

[32] Singla A Sharma U Makkar A Rare metastatic sites of renal cell carcinoma: a case series Pan Afr Med J 2022 42 26 35910051 10.11604/pamj.2022.42.26.33578 PMC9288148

[33] Wallace J Abelardo E Ramachandran K Prabhu V Renal cell carcinoma uvula metastasis leading to airway compromise: an unusual site BMJ Case Rep 2022 15 04 e248098 10.1136/bcr-2021-248098 PMC8995948 35396238

[34] Liu Y Vargo R J Bilodeau E A Analytic survey of 57 cases of oral metastases J Oral Pathol Med 2018 47 03 275 280 29283437 10.1111/jop.12672

[35] Abraham G P Cherian T Mahadevan P Avinash T S George D Manuel E Detailed study of survival of patients with renal cell carcinoma in India Indian J Cancer 2016 53 04 572 574 28485354 10.4103/0019-509X.204758

[36] Howlander N Noone A M Krapcho M SEER cancer statistics review 1975-2016Natl. Cancer Institute2019. Accessed February 5, 2024 at:https://seer.cancer.gov› csr › 1975_2016

[37] Tsivian M Moreira D M Caso J R Mouraviev V Polascik T J Cigarette smoking is associated with advanced renal cell carcinoma J Clin Oncol 2011 29 15 2027 2031 21502558 10.1200/JCO.2010.30.9484

[38] Wyszyńska-Pawelec G Gontarz M Zapała J Szuta M Minor salivary gland tumours of upper aerodigestive tract: a clinicopathological study Gastroenterol Res Pract 2012 2012 780453 22675346 10.1155/2012/780453 PMC3364546

[39] Hyams V J Differential diagnosis of neoplasia of the palatine tonsil Clin Otolaryngol Allied Sci 1978 3 02 117 126 668170 10.1111/j.1365-2273.1978.tb00674.x

[40] Moch H Amin M B Berney D M The 2022 World Health Organization Classification of Tumours of the Urinary System and Male Genital Organs - Part A: Renal, penile, and testicular tumours Eur Urol 2022 82 05 458 468 35853783 10.1016/j.eururo.2022.06.016

